# Synthesis and Characterization of Lignin‐Based Polycarbonate Polyols for Flexible Polyurethane Foam Application

**DOI:** 10.1002/cssc.202502528

**Published:** 2026-01-29

**Authors:** Enoch Kofi Acquah, Daniel Holmes, Kevin Dunne, Anibal Bher, Saeid Ansari Sadrabadi, Amin Joodaky, Rafael Auras, Mojgan Nejad

**Affiliations:** ^1^ Chemical Engineering and Materials Science Department Michigan State University East Lansing Michigan USA; ^2^ Department of Chemistry Michigan State University East Lansing Michigan USA; ^3^ School of Packaging Michigan State University East Lansing Michigan USA; ^4^ Department of Forestry Michigan State University East Lansing Michigan USA

**Keywords:** flexible foam, lignin polycarbonate polyol, oxyalkylation, polyurethane, transesterification

## Abstract

With the rising demand for sustainable materials, lignin‐based polyols offer a promising renewable alternative to traditional petroleum‐based polyols in flexible polyurethane (PU) foams. This study focuses on synthesizing novel high‐performance lignin‐based polycarbonate polyols via transesterification with dimethyl carbonate. The resulting lignin polyols exhibited hydroxyl values ranging from 111 to 179 mg KOH/g and viscosities of 12,000–26,000 mPa·s, thereby enhancing the suitability of lignin for flexible foam formulation. An in‐depth structural analysis using proton, carbon, phosphorus, and 2D nuclear magnetic resonance confirmed the grafting of long polyether chains and the introduction of multiple carbonate linkages onto the lignin structure. Foams were formulated by replacing up to 60% of petroleum‐based polyols with either synthesized lignin polyol or a mixture of lignin and soy polyols. Formulated foams demonstrated superior mechanical properties, including enhanced tensile strength and load‐bearing capacity, compared to petroleum‐based foams. Additionally, the developed foams with biobased polyols exhibited improved thermal stability, shock absorption, and partial biodegradability.

## Graphical abstract

Lignin was converted into polycarbonate polyols through oxyalkylation with propylene carbonate, followed by transesterification with dimethyl carbonate. The resulting biobased polyols were used directly to formulate flexible polyurethane foams for automotive seating applications. The figure (graphical Abstract) is attached.

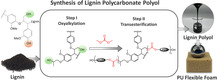

## 1 Introduction

Polyurethane (PU) flexible foams are widely used in various industries due to their versatility, lightweight nature, and excellent mechanical properties [1]. The performance of PU flexible foams is heavily influenced by the type of polyol used in their synthesis, with polycarbonate polyols emerging as a superior alternative to traditional polyester and polyether polyols [2]. Polycarbonate polyols offer enhanced mechanical strength [3, 4, 5], superior resistance to hydrolysis [6], improved thermal stability [3, 7, 8], and impact resistance [9], making them particularly suitable for higher‐end applications such as automotive seating. In contrast, polyester polyols, though mechanically strong and resistant to oxidation, are prone to hydrolysis [10]. Polyether polyols, despite their hydrolytic stability, often lack the mechanical strength and thermal resistance provided by polycarbonate polyols [2]. Consequently, polycarbonate polyols have received considerable attention as a high‐performance alternative for flexible PU foam formulations. However, their relatively high cost limits their usage in flexible PU foam applications.

Traditionally, the synthesis of polycarbonate polyols relied on phosgene‐based processes that, while effective, raised significant safety and environmental concerns due to phosgene's toxicity [2]. Developing safer and more sustainable alternatives, such as dimethyl carbonate (DMC), has revolutionized the production of polycarbonate polyols [11]. DMC is a nontoxic, biodegradable reagent that can be derived from renewable resources, including carbon dioxide captured from the atmosphere and methanol, making it a greener option [12]. Unlike phosgene, DMC does not require stringent safety measures during handling, enabling safer and more efficient polycarbonate synthesis. Additionally, DMC promotes the introduction of carbonate linkages into polymer chains, enhancing the mechanical and thermal properties of the resulting polyols while maintaining processability [13].

Several studies have demonstrated the effectiveness of DMC in polycarbonate polyol production using 1,4 butanediol [14, 15], 1,6‐hexanediol [14, 15], and isosorbide [16]. Foy et al. [17] synthesized linear aliphatic polycarbonate macro glycols using DMC and diols, showing that DMC could produce well‐defined polyols with tailored molecular weights and functional properties suitable for PU applications. Similarly, Song et al. [14] prepared polycarbonate diols (PCDLs) through the reaction of DMC with diols using a KNO_3_/γ‐Al_2_O_3_ catalyst, demonstrating precise control over molecular weight and polymer architecture, which is critical for optimizing PU flexible foam properties. Further advancements in DMC‐based polycarbonate synthesis were made by Kim et al. [3], who developed carbonate‐type macrodiols via base‐catalyzed polycondensation of DMC with co‐diols. Their study demonstrated an environmentally friendly approach to macrodiol synthesis, leading to transparent, self‐healing thermoplastic polyurethanes. Organo catalysts such as guanidines and amidines have also been explored for the synthesis of polycarbonate polyols due to their high catalytic activity and, in some cases, their bifunctional ability to activate both diols and diethyl carbonate, making them ideal catalysts [15]. Collectively, these studies establish DMC‐based synthesis as a promising route for producing high‐performance polyols with tunable properties for polyurethane applications [3, 14, 17]. Despite these advancements, limited effort has been made to incorporate biobased components in polycarbonate polyols. To date, the few reported biobased PCDLs in literature have primarily been derived from isosorbide [16, 18], highlighting a significant opportunity for further development.

Notably, the integration of lignin, a renewable and aromatic biopolymer, into the synthesis of polycarbonate polyols remains unexplored. Lignin's rich hydroxyl functionality and rigid structure offer a unique opportunity to enhance the sustainability and performance of polycarbonate polyols. Research on the use of lignin in flexible PU foams remains relatively limited, with only about 17 studies published to date [19, 20, 21, 22, 23, 24, 25, 26, 27, 28, 29, 30, 31, 32, 33, 34, 35]. However, these studies demonstrate that lignin can significantly improve the thermomechanical properties of PU foams [19, 20, 21, 22, 23, 24, 25, 26, 27, 28, 29, 30, 31, 32, 33, 34, 35]. Therefore, incorporating lignin in polycarbonate polyol synthesis could provide an avenue to leverage the unique structural and functional properties of lignin while reducing the reliance on petroleum‐based raw materials. Additionally, utilizing lignin, which is produced as a byproduct from pulp and paper industries, could open up an opportunity to reduce the high cost of conventional polycarbonate polyols, thus expanding its application in flexible PU foam. This is the first study to synthesize lignin‐based polycarbonate polyols and evaluate their suitability for formulating flexible PU foams.

## 2 Materials and Methods

### 2.1 Materials

Huntsman Corporation generously supplied polyether polyol (MW 6000 Da, hydroxyl value 28 mg KOH/g), propylene carbonate, polymeric methylene diphenyl diisocyanate (NCO content 31.5%, functionality‐2.7), gelation catalyst (33% triethylenediamine), and blow catalyst (70% bis‐(2‐dimethylaminoethyl ether solution). Momentive Performance Materials provided siloxane surfactants, while Cargill supplied soy polyol (BIOH 2828). Analytical reagents, including cyclohexanol (99% purity), pyridine (HPLC grade), and 2‐chloro‐4,4,5,5‐tetramethyl‐1,3,2‐dioxaphospholane (TMDP), were purchased from Sigma–Aldrich. Additionally, chromium (III) acetylacetonate, deuterated chloroform, HPLC‐grade tetrahydrofuran, DMC (99% purity), potassium carbonate, polyethylene glycol, and acetic anhydride were obtained from Fisher Scientific. Tokyo Chemical Industry (TCI) supplied 1,8‐diazabicyclo [5.4.0]undec‐7‐ene (DBU). Acid hydrolysis of hardwood lignin samples was obtained from Sweetwater Energy, with their properties detailed in Tables S1 and S2 (see Supporting Information).

### 2.2 Polyol Synthesis

#### 2.2.1 Step 1: Propylene Carbonate (PC) Oxyalkylation

Oven‐dried acid hydrolysis hardwood lignin was mixed with two equivalent molar ratios of PC (with respect to the total hydroxyl value of lignin) in a Model 4524 Parr Reactor (0.6 L capacity). Polyethylene glycol (molecular weight 200 Da) was added to the mixture to obtain 30% lignin content. 1,8‐Diazabicyclo [5.4.0] undec‐7‐ene (0.05 molar equivalent of the total hydroxyl value of lignin) was added to the mixture. The reactor was purged with nitrogen for 5 min to completely remove air trapped in the reactor. The mixture was heated at 150°C for 1.5 h, mixing at 400 rpm. The gas outlet valve was opened occasionally to vent carbon dioxide produced from the reaction to avoid pressure build‐up in the reactor. The reaction mixture was allowed to cool to room temperature before the transesterification step was carried out.

#### 2.2.2 Step 2: DMC Transesterification

The mixture obtained from the oxyalkylation step was heated with 0.5 equiv of DMC (based on the total hydroxyl value of the polyol mixture in step 1) at 150°C under reflux for 30 min. At the end of the reaction, methanol and unreacted DMC were removed completely at 90°C under reduced pressure using a vacuum pump. The process of polyol synthesis was repeated for polyethylene glycol with molecular weights of 400, 600, and 1000 Da.

### 2.3 Polyol and Foam Characterization

Polyol characterization included determination of hydroxyl value (OHV), viscosity, modified lignin content, and molecular structure. OHV was measured using ^31^P nuclear magnetic resonance (NMR) spectroscopy, while viscosity was determined using a hybrid rheometer at a constant shear rate (50 s^−1^). Modified lignin content was estimated gravimetrically. Structural analysis was conducted via Fourier Transform Infrared Spectroscopy (FTIR), ^1^H, ^13^C, (Heteronuclear Single Quantum Coherence) HSQC, and (Heteronuclear Multiple Bond correaltion (HMBC) NMR techniques. Differential scanning calorimetry (DSC) was used to determine the glass transition temperature (*T*_g_) of lignin samples.

Flexible PU foams were formulated by partially replacing petroleum‐based polyols with synthesized lignin polyol (up to 40%) and, in some cases, in combination with soy polyol (up to 60% substitution). The resulting foams were characterized for physical and mechanical properties, including density, compressive force deflection (CFD), support factor, tensile strength, elongation at break, tear strength, and hysteresis loss, following ASTM D3574 using an Instron universal testing machine. Foam morphology was examined by scanning electron microscopy (SEM), and shock absorption was assessed using a custom drop‐test apparatus (impact tester) to generate cushion curves. Thermogravimetric analysis (TGA) was performed to evaluate the thermal stability of PU foams. Biodegradation of selected PU foam samples was evaluated under controlled composting conditions over 120 days, with % mineralization used to assess the extent of degradation. Detailed experimental procedures, equipment specifications, and supplementary data are provided in the Supporting Information.

## 3 Results and Discussion

### 3.1 Chemical Structural Analysis

**SCHEME 1:**
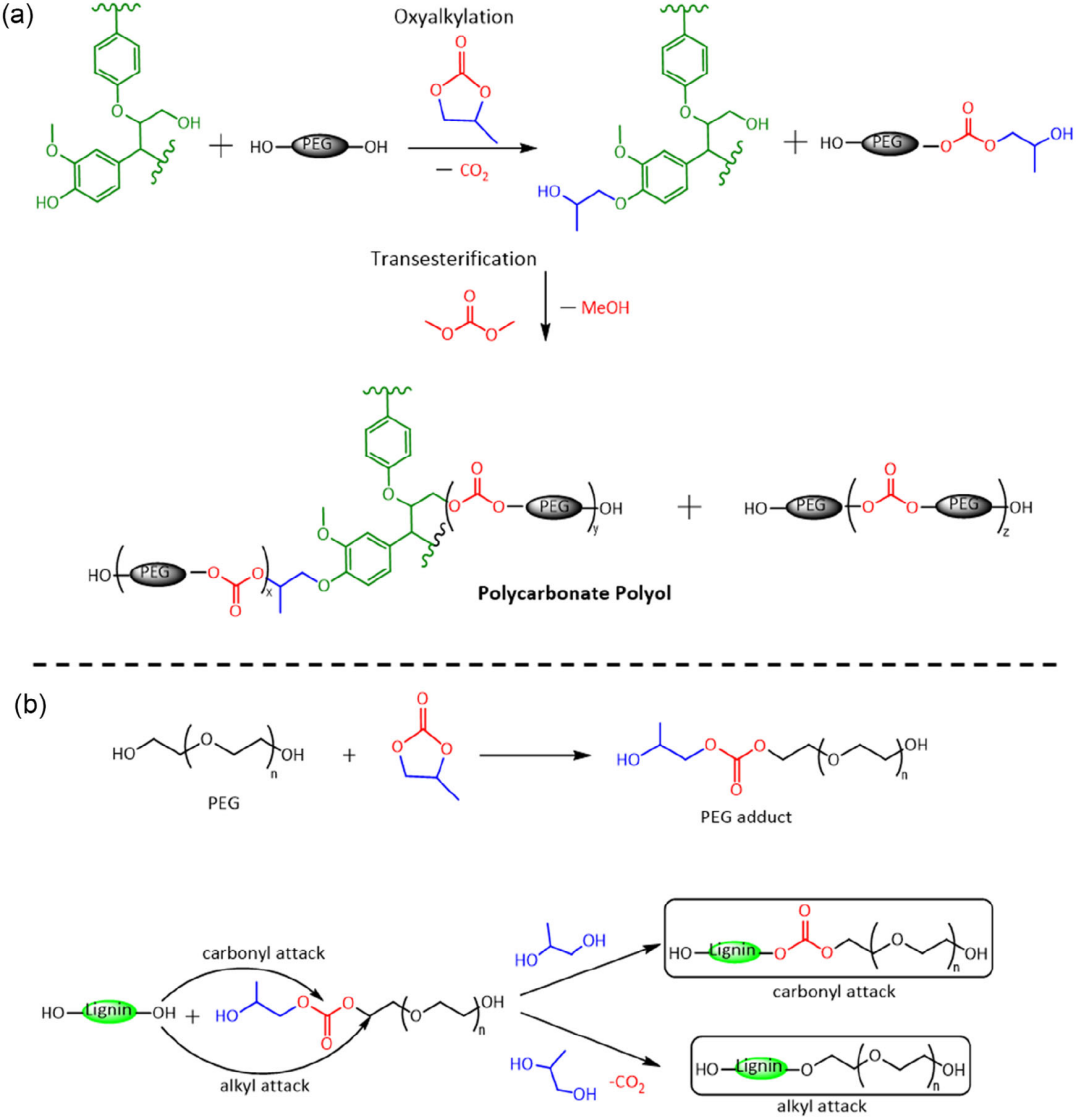
Synthesis of lignin‐based polycarbonate polyol via (a) PC oxyalkylation and DMC transesterification reaction. (b) Schematic representation of the possible reaction mechanism leading to the grafting of PEG on lignin in the PC oxyalkylation reaction step.

A comprehensive structural analysis was performed on the synthesized lignin polyol during both the PC oxyalkylation and DMC transesterification steps (Scheme 1a). This analysis offered deeper insights into the reaction mechanisms and confirmed the configuration of repeating units in the resulting polycarbonate polyol. NMR analysis of the crude lignin polyol after the oxyalkylation reaction indicated that it was composed of oxyalkylated lignin, polyethylene glycol (PEG) and its adduct (formed from the reaction between PEG and PC), unreacted PC, and some propylene glycol (PG). It also verified the reaction between PEG and DMC, demonstrating the successful introduction of carbonate linkages and a significant reduction in total hydroxyl content. Detailed information on the NMR analysis of the lignin polyol is provided in the Supporting Information. Although NMR provided detailed information on structural changes in PEG, its resolution was insufficient to reliably assess modifications to the lignin backbone due to the complex mixture in the crude material. As a result, lignin precipitation was necessary to isolate and further investigate the structural changes.

**FIGURE 1:**
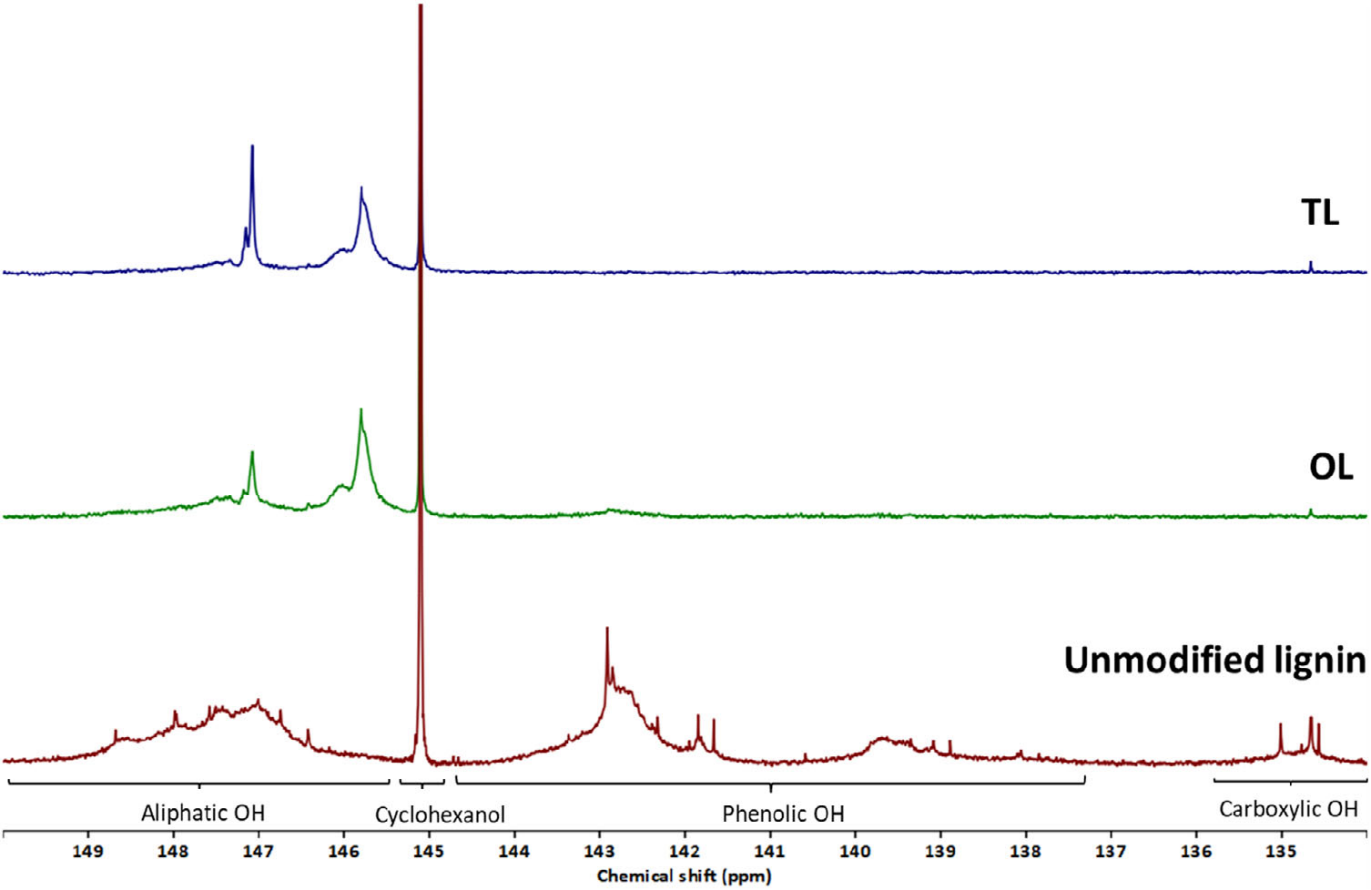
Expansion of a ^31^P NMR spectra of unmodified, precipitated PC oxyalkylated (OL), and DMC transesterified (TL) lignin. Samples were phosphitylated prior to analysis.

The precipitation of modified lignin fractions (oxyalkylated lignin (OL) and trans‐esterified lignin (TL)) was crucial for gaining clearer insight into the structural changes of lignin during PC oxyalkylation and DMC transesterification reactions. ^31^P NMR analysis of precipitated OL, as shown in Figure 1, revealed a significant reduction in the phenolic (137–144.6 ppm) and carboxylic acid (134–135.9 ppm) hydroxyl groups of lignin. This was expected, as these groups reacted with PC. Interestingly, a primary aliphatic hydroxyl group from PEG was also detected around 147 ppm, suggesting a possible PEG grafting onto lignin. This peak further intensified after the transesterification reaction, indicating additional incorporation of PEG on the lignin structure.

Notably, a small amount of phenolic hydroxyl groups (0.18 mmol/g) remained in OL but was undetectable after transesterification. Their absence in TL indicates that the lignin‐PC reaction may have continued during the transesterification step. Due to the acidic nature of phenolic OH groups, it favors the reaction with PC; however, it is also possible to react with DMC. Overall, the total hydroxyl value (OHV) decreased from 5.43 mmol/g in unmodified lignin to 3.61 mmol/g in OL and further to 2.88 mmol/g in TL, demonstrating the progressive modification of lignin's hydroxyl functionalities, making it more suitable for PU flexible foam and some coating applications.

**FIGURE 2:**
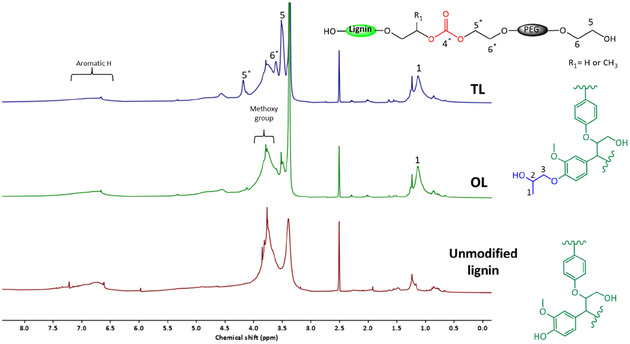
Expansions of ^1^H NMR spectra of unmodified, precipitated PC oxyalkylated (OL), and DMC transesterified (TL) lignin.

^1^H NMR analysis of OL, as presented in Figure 2, showed a new broad methyl proton signal at 1.13 ppm, which was absent in unmodified lignin. This signal is attributed to methyl protons from the hydroxypropyl units grafted onto lignin (peak 1), formed through the reaction of lignin's hydroxyl groups with PC. Additionally, a terminal methylene proton signal was detected in OL around 3.5 ppm which may have originated from grafted PEG during the oxyalkylation reaction.

**FIGURE 3:**
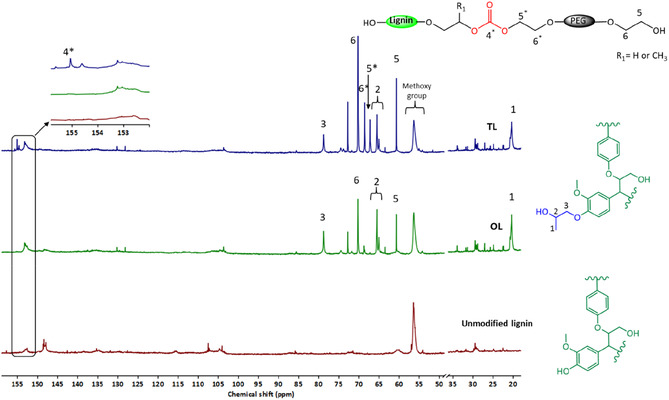
Expansion of ^13^C NMR spectra of unmodified, precipitated PC oxyalkylated (OL), and DMC transesterified (TL) lignin.

The success of the oxyalkylation reaction was further confirmed by the appearance of hydroxypropyl carbon signals in the ^13^C NMR spectrum of OL at 20.5, 65.2, and 78.8 ppm (Figure 3). The absence of the carbonyl carbon signal around 154 ppm in OL indicates that the aliphatic hydroxyl groups in lignin did not participate in the reaction with PC as they tend to react with the carbonyl carbon over that of the alkylene carbon. Instead, the reaction selectively targeted more acidic functional groups, such as carboxylic and phenolic hydroxyl groups, consistent with similar findings in the literature [36]. Kuhnel et al. [37] also reported that the reaction between lignin's aliphatic hydroxyl groups and PC to form carbonate linkages can be achieved to some extent under high PC concentrations, prolonged reaction times, and the presence of a potassium carbonate catalyst.

Three new methylene carbon signals at 60.6 ppm (peak 5), 70.2 ppm (peak 6), and 72.8 ppm, consistent with a long polyether PEG‐like chain, were detected in the ^13^C NMR spectrum of OL, aligning with observation from the ^31^P NMR spectrum. These signals persisted even after multiple washings of OL with distilled water. Since this reaction was performed in the presence of PEG, it's likely that PEG was incorporated into OL. Indeed, when the oxyalkylation reaction was repeated without PEG, these methylene carbon signals were absent, as seen in Figure S1 (see Supporting Information), confirming that their presence was directly linked to the inclusion of PEG in the reaction.

Scheme 1b shows a schematic representation of the proposed reaction mechanism responsible for PEG grafting onto lignin during the oxyalkylation step. In summary, the formation of this structure likely involves the following steps: (a) PEG reacts with PC, resulting in a PEG adduct with a carbonate linkage, terminated by a hydroxyl group. (b) Lignin hydroxyl groups react with the PEG adduct, either at the carbonyl site or the alkyl site. A carbonyl attack, likely initiated by aliphatic hydroxyl groups, leads to the formation of a carbonate linkage, with PG as the leaving group. An alkyl attack, likely involving phenolic hydroxyl groups, produces an ether linkage, with PG and carbon dioxide as byproducts. The high intensity of the methylene carbon signal around 70 ppm and the absence of carbonyl carbon around 155 ppm in OL in Figure 3 suggests that the alkyl attack was the predominant reaction pathway, resulting in the grafting of PEG onto lignin during the oxyalkylation step. Additionally, the presence of PG in the oxyalkylation reaction mixture further substantiates the proposed reaction mechanism.

The inclusion of PEG provides an additional reactant for PC, enhancing its consumption beyond what occurs through lignin oxyalkylation reaction and reducing the amount of unreacted PC in the lignin polyol. PEG was chosen as the co‐polyol due to its capacity to solubilize lignin, and the fact that its biobased alternative is commercially available. Furthermore, the incorporation of PEG into lignin's structure during oxyalkylation presents a great opportunity to reduce lignin's inherent rigidity. The linear polyether chains in PEG impart flexibility, which is essential for formulating flexible PU foam. Also, due to the primary aliphatic hydroxyl group present in the grafted PEG, it further helps facilitate lignin's reaction with DMC.

**FIGURE 4:**
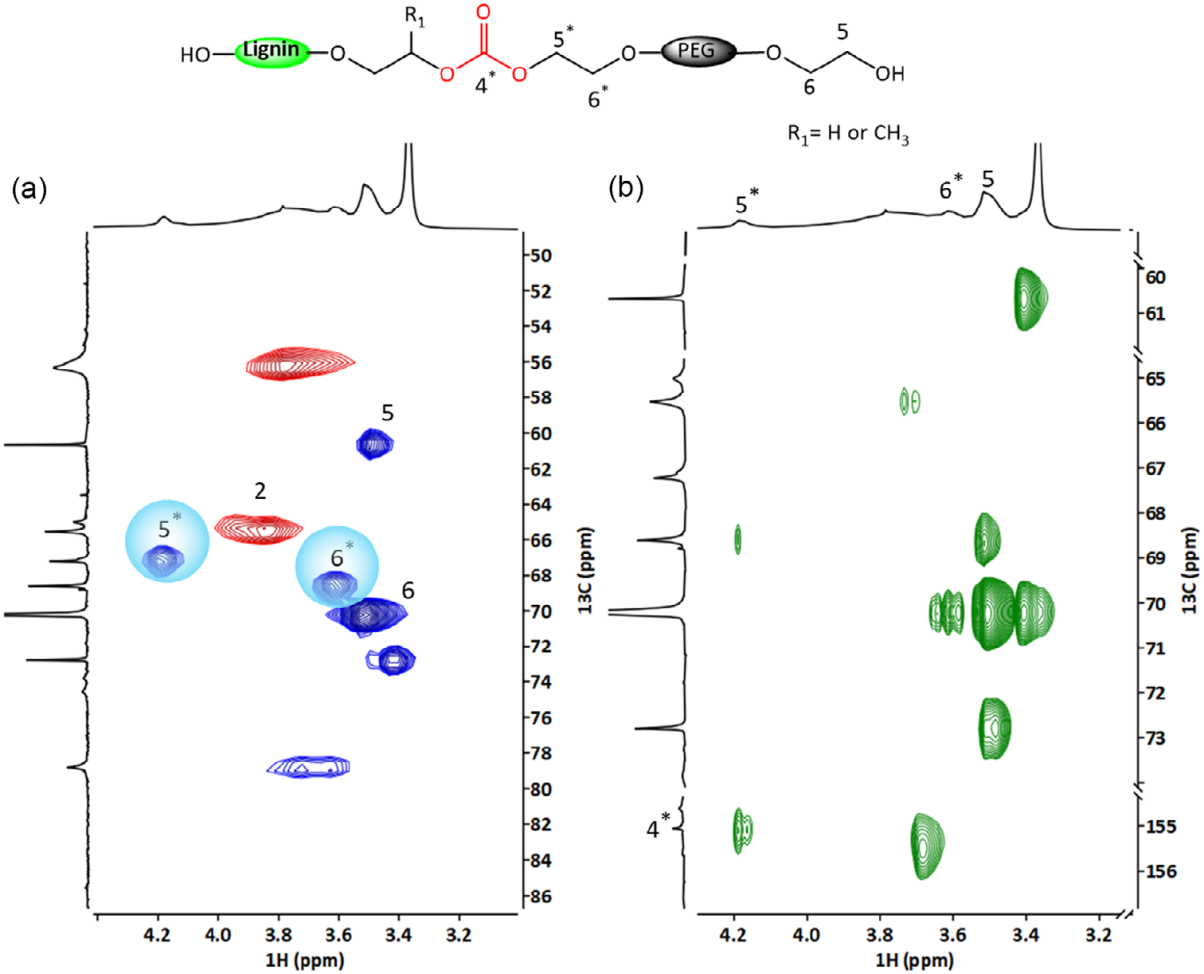
Expansion from (a) HSQC and (b) HMBC NMR spectra of precipitated DMC transesterified (TL) lignin.

Analysis of TL revealed the emergence of two distinct methylene proton peaks at 3.61 ppm (peak 6*) and 4.18 ppm (peak 5*) in the ^1^H NMR spectra (Figure 2), which are directly bonded to carbons at 68.70 and 67.1 ppm, respectively, as identified in the HSQC spectra (Figure 4a). ^13^C NMR analysis (Figure 3) showed a new carbonyl carbon peak at 155.1 ppm (peak 4*). This peak has a cross‐peak with methylene protons at 4.18 ppm (peak 5*), as seen in the HMBC spectra (Figure 4b), thereby confirming incorporation of carbonate linkages in TL. The methylene proton at 4.18 (peak 5*) ppm also has a cross‐peak with a methylene carbon at 68.70 ppm, indicating that a PEG chain has been grafted onto the lignin macromolecule via a carbonate linkage. Additionally, the increased intensity of the methylene carbon signal at 60.6 ppm (peak 5), associated with the terminal CH_2_ group adjacent to a primary hydroxyl group, and the methylene carbon at 70.2 ppm (peak 6) further support the successful grafting of PEG via the transesterification reaction. Notably, there was no cross‐peak between the methine proton of OL at 3.79 ppm and the carbonate carbonyl at 155.1 ppm, indicating that the hydroxypropyl units in OL did not participate in the transesterification reaction. This implies that secondary aliphatic hydroxyl groups were less reactive toward DMC under current reaction conditions. This is likely due to steric hindrance provided by the methyl pendant group.

Differential scanning calorimetry (DSC) analysis, summarized in Table 1, provided additional evidence of structural modifications. The glass transition temperature (*T*_g_) of lignin decreased from 156°C to 109°C after oxyalkylation, representing a 30% reduction. This reduction is attributed to grafting hydroxypropyl units and PEG onto the lignin structure. The *T*_g_ further decreased to 62°C after the transesterification step due to the incorporation of additional long, flexible polyether PEG chains. Depending on the molecular weight of the PEG used, the *T*_g_ of OL ranged from 88°C to 109°C, while the *T*_g_ of TL ranged from 60°C to 65°C. Notably, PEG molecular weight had the greatest impact during the oxyalkylation step, with higher molecular weights yielding lower *T*_g_ values. These findings highlight the potential for tailoring lignin's thermal properties for applications requiring lower *T*_g_ values, such as PU coatings and flexible foams.

**TABLE 1:** Glass transition temperature (*T*_g_) of precipitated PC oxyalkylated (OL) and DMC transesterified (TL) lignin based on the molecular weight (MW) of polyethylene glycol (PEG) employed in the polyol synthesis. TL_PEG1000_ resulted in a gelled polymer. *T*_g_ (unmodified lignin) = 160 ± 5.5°C.

PEG MW, Da	OL *T*_g_, °C	TL *T*_g_, °C
200	106 ± 3.8^a^	62 ± 2^a^
400	109 ± 6.0^a^	60 ± 2^a^
600	89 ± 0.9^b^	65 ± 4^a^
1000	96 ± 1.1^b^	—

*Note*: Within each column, values sharing the same superscript letter (a,c) are not significantly different, whereas values with different letters are significantly different (*p* ≤ 0.05, Tukey's test).

### 3.2 Lignin Polyol Properties

The transesterification process involving oxyalkylation derivatives and DMC was significantly influenced by both reaction time and the quantity of DMC used. Initial experiments utilizing 1 equivalent of DMC at 150°C for 1 h yielded a highly viscous gel‐like polymer that could not be used in foam formulations. This outcome was anticipated due to lignin's high functionality, which could lead to crosslinking with PEG or another lignin chain, resulting in the formation of high molecular weight polycarbonate polyols. Even when the reaction time was reduced to 0.5 h while keeping the temperature (150°C) and DMC (1 eq.) quantity constant, the resulting materials exhibited significantly high viscosities. However, by decreasing the amount of DMC to 0.5 equivalent and maintaining a reaction time of 0.5 h, a product with a workable viscosity (11,660–25,950 mPa⋅s) was obtained, suitable for further characterization and foam formulation. This indicated the sensitivity of transesterification reaction to both reaction time and the amount of DMC. In addition, PEG with various molecular weights (200, 400, 600, and 1000 g mol^−1^) was used to prepare polycarbonate polyols with lower hydroxyl values. The properties of synthesized lignin polyols (Ligols) are summarized in Table 2. It was observed that an increase in the molecular weight of PEG led to a reduction in the total hydroxyl value. This result was expected as longer chains, such as PEG 1000 (112.2 mg KOH/g), tend to have a much lower hydroxyl value than PEG 200 (561 mg KOH/g).

**TABLE 2:** Modified lignin content, hydroxyl value, viscosity after PC oxyalkylation and hydroxyl value, percent hydroxyl value reduction, and viscosity of lignin polyol after DMC transesterification reaction based on PEG molecular weight employed.

Lignin Polyol ID	PEG Mw	Oxyalkylation			Transesterification		
		Modified lignin content, %	Hydroxyl value, mg KOH/g	Viscosity, mPa⋅s	Hydroxyl value, mg KOH/g	Hydroxyl value reduction, %	Viscosity, mPa⋅s
Ligol A	200	39 ± 2.1^a^	365	2600 ± 30^a^	179	50.96	11,660 ± 450^a^
Ligol B	400	35 ± 1.8^a^	266	7550 ± 10^b^	151	43.23	16,880 ± 750^b^
Ligol C	600	38 ± 2.0^a^	205	13,790 ± 430^c^	111	45.85	25,950 ± 930^c^
Ligol D	1000	36 ± 1.1^a^	157	30,890 ± 500^d^	—	—	—

*Note*: Within each column, values sharing the same superscript letter (a–d) are not significantly different, whereas values with different letters are significantly different (*p* ≤ 0.05, Tukey's test).

[a] Ligol A, B, and C, after the transesterification step, were directly used to formulate foams.

[b] Ligol D yielded a gelled lignin polyol after the transesterification reaction.

Using low‐molecular‐weight PEGs (200–400 g mol^−1^) in the oxyalkylation step produced lignin polyols with hydroxyl values of 205–365 mg KOH/g and relatively low viscosities (2600–13,790 mPa·s). These values are notably lower than those reported by Duval et al. [38], who synthesized oxyalkylated lignin polyols using comparable PEG molecular weights (150–400 g mol^−1^) but obtained substantially higher hydroxyl values (380–600 mg KOH/g) and viscosities (10,000–40,000 mPa·s) at the same lignin loading (30%). The more favorable hydroxyl values and viscosities observed in our study are likely attributable to differences in reaction conditions, particularly the use of DBU instead of potassium carbonate and significantly shorter reaction times (1.5 h). Longer reaction times (4–6 h) and a potassium carbonate catalyst have been reported to promote lignin chain‐coupling side reactions [37], which, in turn, increase both hydroxyl value and viscosity. Although PEG 1000 yielded a polyol with a hydroxyl value within the acceptable flexible foam polyol range (157 mg KOH/g) following the oxyalkylation process, it exhibited excessively high viscosity (30,890 mPa⋅s) and incomplete phenolic OH conversion. This might be attributed to the elevated viscosity of the medium, which may have restricted the reaction rate. The presence of phenolic hydroxyl groups in modified lignin is undesirable because of their low reactivity with isocyanates [39]. Hence, PEGs with lower molecular weights (≤600 Da) are more suitable for this application, as they yielded polycarbonate polyols with a moderately low hydroxyl value (111–179 mg KOH/g) and workable viscosities (≤25,000 mPa⋅s), ideal for PU flexible foam formulation.

### 3.3 Foam Properties

#### 3.3.1 Mechanical Properties

**FIGURE 5:**
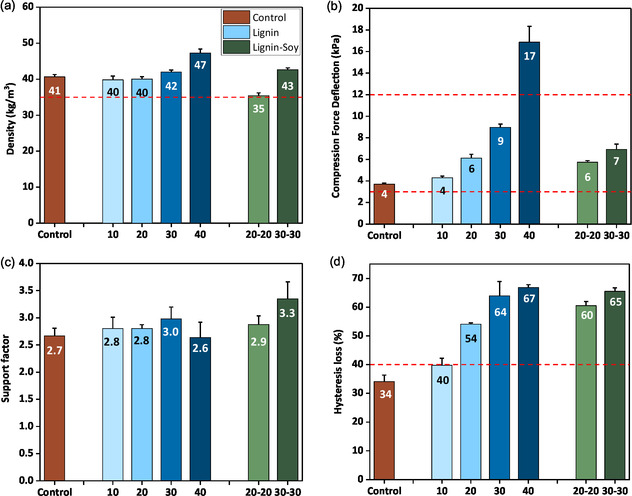
Mechanical properties of lignin polyol (LP)‐based PU flexible foams (up to 40% petroleum‐based polyol replacement) and lignin‐soy polyol (LP‐SP) foams as measured by ASTM D3574 test method: (a) density, (b) compression force deflection at 50% strain, (c) support factor, and (d) hysteresis loss. All lignin‐based foams were formulated with Ligol A.

**FIGURE 6:**
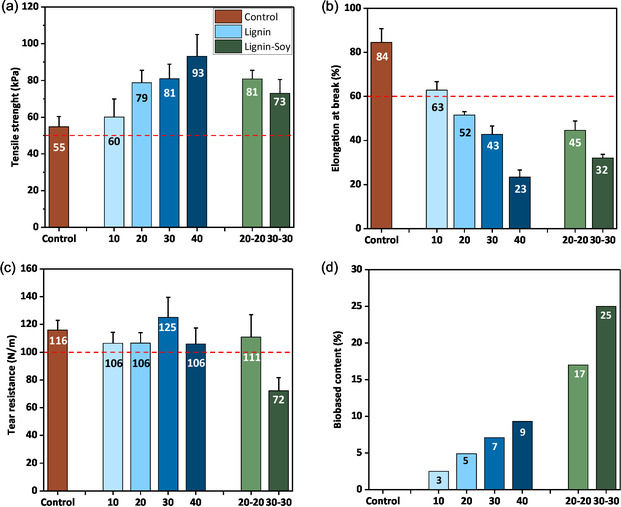
ASTM D3574 measured (a) tensile strength, (b) ultimate elongation at break, (c) tear strength, and (d) calculated biobased content of developed lignin polyol (LP)‐based PU flexible foams (up to 40% petroleum‐based polyol replacement) and lignin‐soy polyol (LP‐SP) foams. All lignin‐based foams were formulated with Ligol A.

**FIGURE 7:**
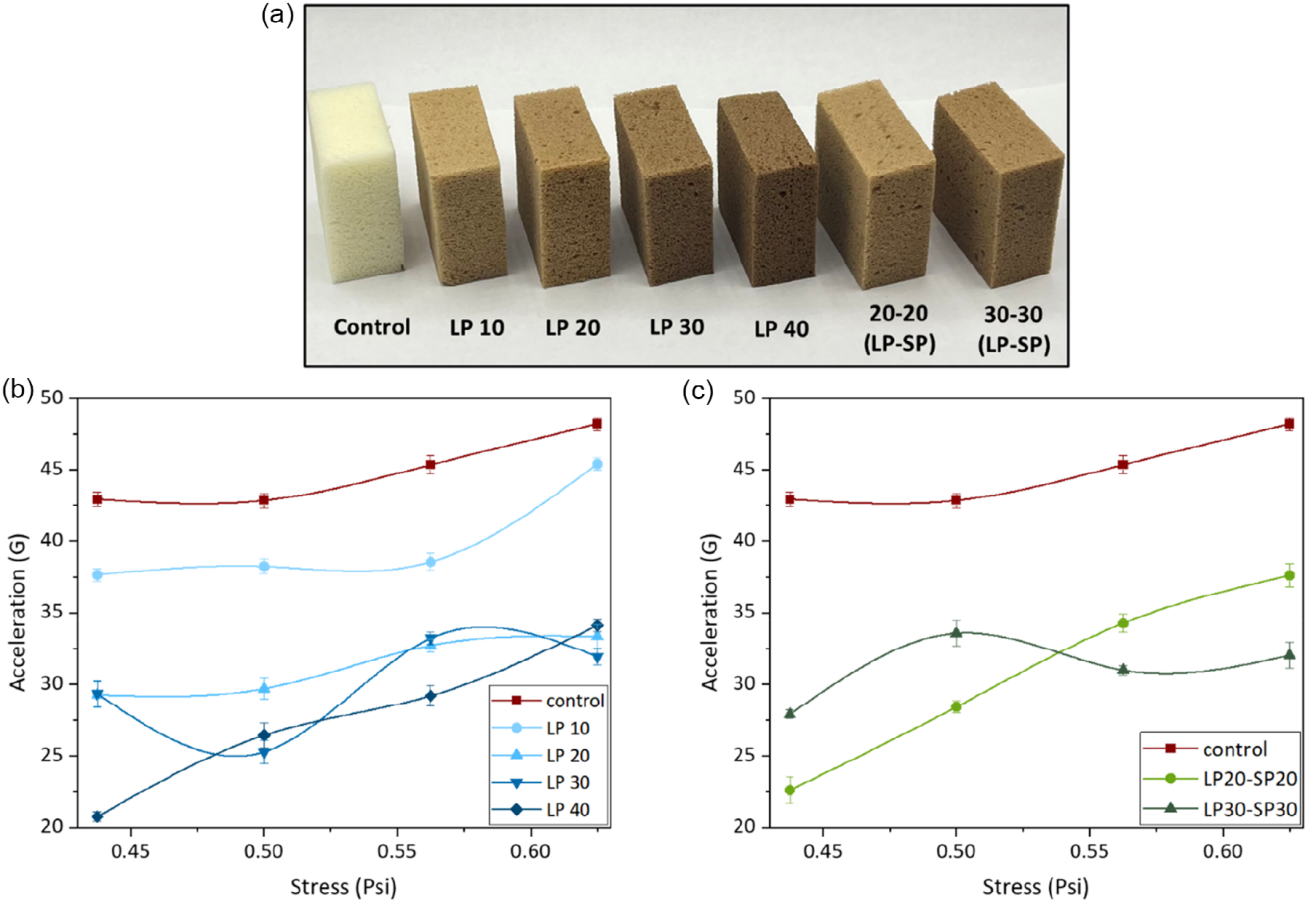
Flexible PU foams produced by replacing (a) up to 40% of petroleum‐based polyols with synthesized lignin polyol (LP) and up to 60% with a combination of LP and soy polyol (SP). Cushion curves of (b) LP‐based PU flexible foams (0%, 10%, 20%, 30%, and 40% petroleum‐based polyol substitution) and (c) LP/SP‐based PU flexible foams. All lignin‐based foams were formulated with Ligol A.

A comparative study on the impact of lignin polyol on flexible PU foam properties was performed by formulating foams with Ligol A (179 mg KOH/g) due to its relatively lower viscosity. Figure 7a shows the flexible PU foams produced by partially replacing petroleum‐based polyols with synthesized lignin polyols. Foam mechanical properties, presented in Figures 5 and 6, were measured according to the ASTM D3574 standard testing method and compared to the standard requirement for automotive seating applications (red border lines) [24, 40]. As the lignin polyol content (in parts per hundred‐gram polyol (pphp)) in the foams increased from 10% to 40%, there was a noticeable increase in the density values. This trend is likely due to the higher levels of DBU (1,8‐diazabicyclo [5.4.0] undec‐7‐ene) catalyst present at higher lignin polyol loadings, which accelerates the gelation reaction and restricts foam expansion [19]. As a result, the foams were unable to blow enough. Despite these changes, all foam densities remained within the standard range of 35–65 kg/m^3^.

The discussion on foam's mechanical properties (Figures 5 and 6) is presented in two parts: first, the impact of varying lignin polyol content (10%–40%) on foam properties is examined, followed by an evaluation of foams incorporating both lignin and soy polyols to enhance biobased content and optimize performance. The CFD, indicating the foam's resistance to deformation under load, varied with the percentage of lignin polyol used, as seen in Figure 5b. Lignin‐based foams generally exhibited higher CFD values compared to control foams, particularly as the lignin content increased. This suggests that lignin imparts greater rigidity and load‐bearing capacity to the foams owing to its aromatic structure. The support factor, which is an indication of foam's cushioning performance, was within 2.6–3.0, thus making it suitable for use in automotive seating applications.

Tensile strength, a measure of the foam's ability to withstand tension, varied depending on the lignin polyol content, as presented in Figure 6a. Foams with higher lignin content demonstrated higher tensile strength due to the robust aromatic structure of lignin and the relatively high hydroxyl value of lignin polyol. Elongation at break, representing the foam's flexibility, also varied with polyol composition. The lignin‐based foams exhibited lower elongation at break values due to their inherent rigidity (aromatic structure) compared to the long aliphatic polyether chain of commercial polyol, which provides more flexibility.

Tear strength, indicative of the foam's resistance to propagating cracks, was also influenced by the lignin polyol concentration (see Figure 6c). Tear strength of lignin polyol foams remained the same as lignin content increased by up to 20%, after which it increased slightly. The maximum tear strength (125 N/m) was recorded at 30% lignin polyol substitution. Beyond 30% replacement, the tear resistance of the foams dropped significantly. The dip in this property was expected due to lignin's crosslinked nature. The decrease in tear values of 40% lignin incorporation could also be attributed to an increased residual PC content in the foam, which may have plasticized it.

Hysteresis loss, related to energy dissipation during cyclic loading, varied with foam composition. Foams with higher lignin polyol content exhibited greater hysteresis loss and thus lower resiliency. This is due to the high rigidity provided by the aromatic structure of lignin and the relatively high hydroxyl value of the lignin polyol (179 mg KOH/g) compared to the petroleum‐based polyol (28 mg KOH/g). The high energy dissipation rate of lignin polyol‐based foams could benefit sound and shock absorption applications where low resilient foams are required. This comparative analysis offers valuable insights into optimizing foam formulations for each specific application, balancing rigidity, flexibility, strength, and energy dissipation.

Soy polyol was added to the lignin‐based foam formulation as an additional strategy to enhance the biobased content of the foams. Soy polyol, derived from epoxidized and ring‐opened soybean oil [41], is a well‐established biobased alternative with desirable reactivity and sustainability benefits. By blending lignin polyol with soy polyol, lignin‐soy foams were formulated to achieve an improved balance between mechanical performance and renewable content (increasing the amount of soy polyol in automotive seating foams beyond 10%). The LP20‐SP20 and LP30‐SP30 foams were designed to systematically evaluate the effect of soy polyol incorporation on the foams’ performance. The goal was to determine whether the inclusion of soy polyol could compensate for any mechanical drawbacks associated with the rigidity of lignin‐based foams while maintaining the advantages of higher biobased carbon content.

As shown in Figures 5 and 6, the mechanical performance of lignin‐soy foams was highly dependent on the soy polyol loading within the foam. At low soy polyol content, as in LP20‐SP20, most mechanical properties remained unchanged compared to the pure lignin polyol‐based foam (LP20). At higher soy polyol content, as in LP30‐SP30, slight reductions were observed in CFD and tensile strength; however, the foams still met the standard requirement for seating application. Hysteresis loss remained unchanged, but tear strength fell below the standard range (≥100 N/m), necessitating the need for further optimization. The decline in tear strength can be attributed to the structural differences between the soy polyol employed and the conventional polyether polyols they replace. Due to its secondary aliphatic hydroxyl groups, soy polyol exhibits lower reactivity toward isocyanates than the primary hydroxyl groups in petrochemical polyether polyols. Additionally, mid‐chain hydroxyl positioning introduces steric hindrance, which limits its ability to react effectively with isocyanates. Another contributing factor is soy polyol's relatively low molecular weight, which reduces elongation at break. The decrease in tear strength observed in soy‐based flexible PU foams have also been reported by previous studies [42]. While the combination of lignin polyol and soy polyol helps increase the soy content to 30%, the adoption of soy polyols with primary hydroxyl groups could offer a promising pathway to further improve foam properties and increase the biobased content in the final foam. Figure 6d presents the calculated biobased content of the developed flexible PU foams. The LP 40 foams exhibited a biobased content of 9%, primarily originating from lignin, while the LP30‐SP30 foams showed a higher biobased carbon content of 25% due to the incorporation of soy polyol. These values could be increased to 20% (LP40) and 35% (LP30‐SP30) by using biobased PEG in the polyol synthesis and selecting lignin polyol with the lowest hydroxyl value (Ligol C) for foam formulations.

Different synthesized lignin polyols (Ligol A, B, and C), each with varying lengths of PEG chains, were assessed by formulating foams containing 30 parts per hundred polyol (pphp) lignin polyols to examine their impact on foam mechanical properties. As shown in Table 3, there was no change in tear strength, but a reduction in CFD as the chain length increased, yet they were still within the standard requirement for seating applications. In addition, it was generally noted that longer PEG chain lengths led to an increase in the support factor, an empirical measure related to the compression modulus that is widely utilized in the industry to gauge foam cushioning. For example, foams with higher support factors are less prone to “sinking in” when subjected to prolonged stress [19]. There was a reduction in foam's tensile strength as the chain length of PEG increased from 200 to 600 Da in the developed lignin polyols. However, the foam's ultimate elongation remained almost the same with an increase in PEG chain length. This outcome was unexpected since low hydroxyl value polyols typically result in reduced crosslinking density, increasing the foam's strain at break. The high functionality of lignin may have also contributed to foam's restricted flexibility. Notably, there was a significant reduction in hysteresis loss with increasing chain length of polyethylene glycol, indicating a potential enhancement in foam resilience, which is pertinent for seating applications.

**TABLE 3:** Mechanical properties of lignin polyol‐based (Ligol A, B, and C) flexible polyurethane foam (30% petroleum‐based polyol substitution) measured according to ASTM D3574 test standard. Ligols A, B, and C were synthesized with PEG 200, 400, and 600, respectively.

Foam Property	Desired range	Unit	Ligol A	Ligol B	Ligol C
Density	40–65	kg/m^3^	41 ± 0.5^a^	47 ± 0.4^b^	49 ± 0.2^c^
Tear strength	100	N/m	125 ± 14^a^	112 ± 9^a^	113 ± 14^a^
CFD	3–12	kPa	9 ± 0.3^a^	8 ± 0.4^b^	6 ± 0.1^c^
Support factor	Higher is better	—	3 ± 0.2^a^	4 ± 0.2^b^	5 ± 1.0^b^
Tensile strength	≥50	kPa	81 ± 8.0^a^	89 ± 8.7^a^	71 ± 3.0^b^
Elongation	≥60	% strain	43 ± 3.9^a^	45 ± 4.6^a^	41 ± 2.0^a^
Hysteresis loss	Lower is better	%	64 ± 4.9^a^	53 ± 1.0^b^	53 ± 2.0^b^

*Note*: Within a row, values with different letters are significantly different at *p* ≤ 0.05 (Tukey's test).

#### 3.3.2 Impact Test

Impact tests were conducted to study the mechanical response of the developed foams (Figure 7a). Figure 7b,c presents all tested cases’ acceleration (*G*) versus static stress. The peak of each pulse was marked by a dot on the line plots, forming what is known as a cushion curve [43]. Almost all cases exhibit a general increasing trend as the drop weight, which is converted to stress, increases. This suggests that heavier impacts led to higher rebound acceleration responses from the foam. The acceleration response of the foams was also dependent on the composition, with lignin‐based foams demonstrating a lower acceleration value compared to control foams without lignin polyol. This indicates that the lignin‐based foams effectively dissipated the energy impact over a longer period, making it a better shock absorption material. In automotive seating applications, flexible polyurethane foams with low acceleration values are critical, particularly for headrests and cushioning areas. Lower acceleration responses help mitigate the risk of head, neck, and spinal injuries in the event of an impact or accident, making lignin‐based foams a promising alternative for improving passenger safety.

Among the lignin‐based foams, as the amount of lignin polyol increases, the acceleration response decreases, as seen in Figure 7b. Additionally, the combination of soy polyol and synthesized lignin polyol reduced the *G* value, as seen in Figure 7c. Depending on the application, a cushion designer can determine the appropriate shock response range and decide the required cushion material and dimensions. For instance, if the acceleration response limit for an application is 40 gs, a 0.5 psi static stress (from a 4 lb weight drop on an 8 sq‐in foam's top area) that produces about 42 gs on a control foam might fail as the 42 gs is larger than 40 gs limit. In such a case, a lower acceleration value option such as LP20, which responds to 30 gs, should be considered.

#### 3.3.3 Foam Morphology

**FIGURE 8:**
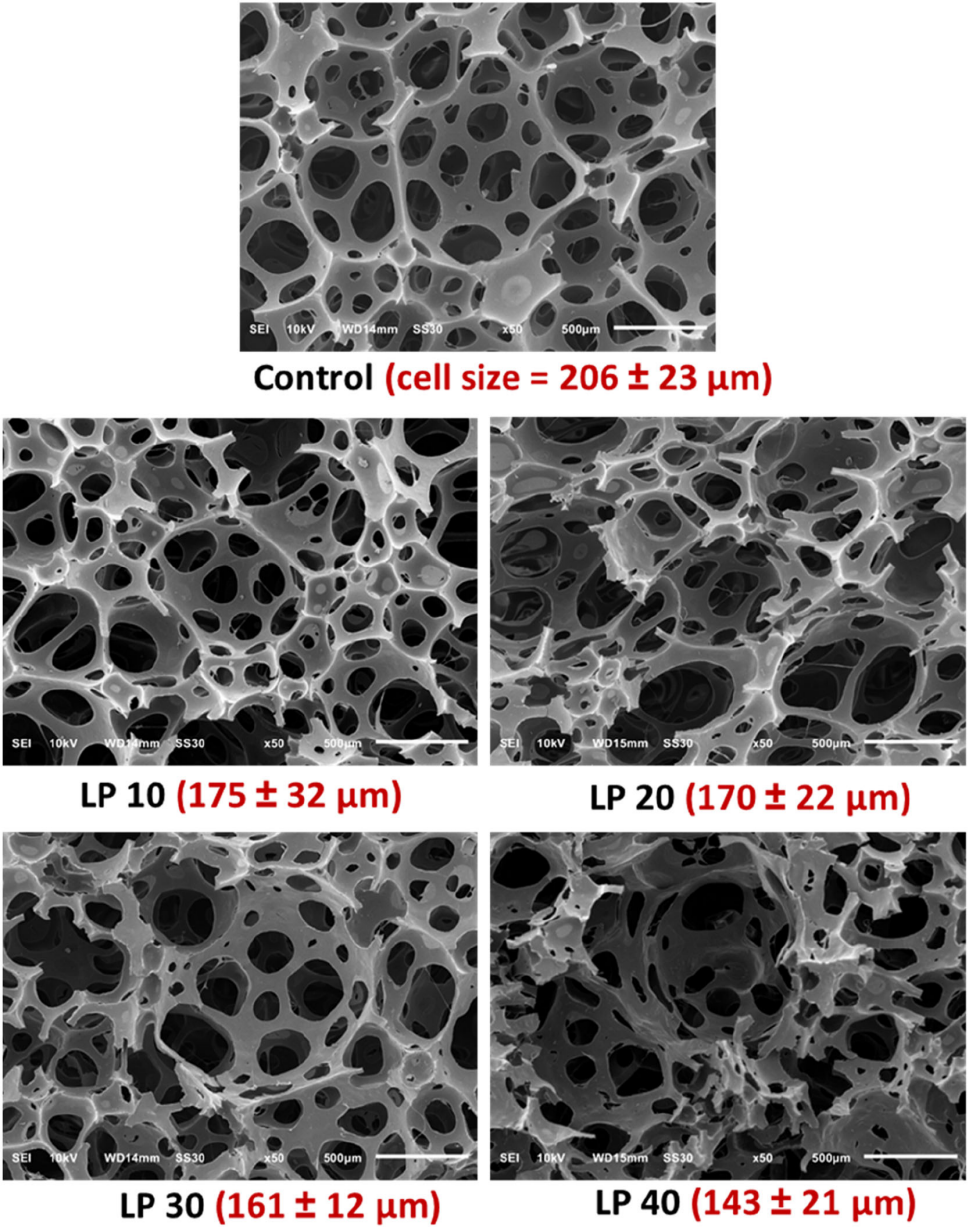
Scanning electron micrographs (SEM) of developed PU flexible foams at 50x magnification, formed by replacing 0%, 10%, 20%, 30%, and 40% of the petroleum‐based polyols with synthesized lignin polyol, LP (Ligol A). Average cell diameter and its standard deviations, µm, are enclosed in parentheses.

Figure 8 shows the SEM images of the developed lignin‐based flexible foams, revealing an open‐cell structure consistent with that of the petroleum‐derived control foam. Unlike flexible polyurethane foams produced using unmodified or solid lignin, no visible lignin particles were observed in the synthesized foams at higher resolution (see Figure S2 in Supporting Information). This absence suggests that the lignin served as a reactive component, integrating into the foam's polymer matrix. This incorporation likely contributed to the superior CFD and tensile properties of the lignin‐based foams compared to the control.

Additionally, the open‐cell sizes of the foams varied with the lignin polyol concentration. The cell diameter decreased as the lignin polyol content increased, a phenomenon attributed to the relatively high hydroxyl value of the synthesized lignin polyols, which enhanced the foam's crosslinking density. Another contributing factor could be the residual DBU present in the synthesized lignin polyols. DBU is known to catalyze gelation reactions [44], and as the lignin polyol concentration rises, so does the level of residual DBU, accelerating the gelation rate and further reducing cell diameter. This effect is consistent with the reactivity data presented in Table S5 (Supporting Information), which shows an apparent decrease in overall foaming time with increasing lignin‐polyol content. This interplay between lignin polyol concentration, hydroxyl value, and residual DBU highlights the intricate relationship between foam formulation and microstructural properties.

#### 3.3.4 Thermal Properties

**FIGURE 9:**
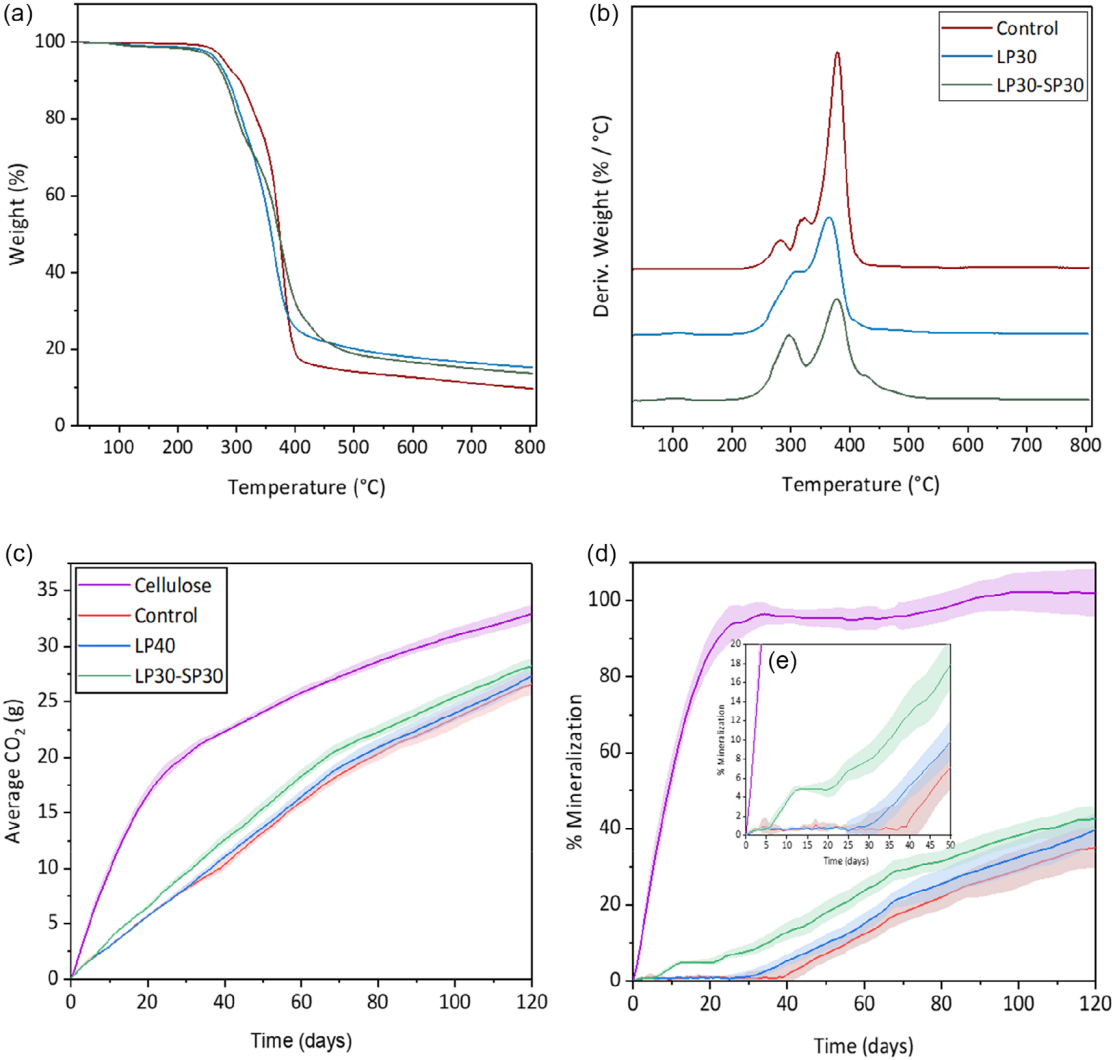
Thermogravimetric analysis (TGA) (a) thermogram, and (b) differential thermogravimetric (DTG) curve (nitrogen environment). Thermophilic anaerobic biodegradation under compost environment (58 ± 2°C and 50 ± 5% relative humidity) (c) CO_2_ evolution until 120 days (d) percentage mineralization until 120 days, and (e) lag phase region of formulated lignin polyol (LP) and soy polyol (SP)‐based flexible PU foams.

Figure 9a,b display the weight loss‐temperature and differential thermogravimetric analysis (DTG) curves of the foams. Both control and lignin polyol‐based foams exhibited similar thermal weight loss characteristics. All foams showed a minor weight loss at temperatures below 150°C, peaking around 105°C, likely due to evaporation of small molecules such as water and PC [45]. As lignin polyol content increases, the percentage of weight loss from small molecule evaporation rises, with LP40 showing the highest weight change at 2.31%. The first major thermal degradation around 296°C–315°C can be associated with weight loss from urethane bond breakage resulting in amines, olefins, and CO_2_ formation [46]. The subsequent weight loss between 354°C and 379°C is linked to soft segment degradation [47]. The soft segment degradation temperature (*T*_max_) decreases with increasing lignin polyol. As seen in Figure S3 (see Supporting Information), the temperature at onset (*T*_onset_) and the temperature at 10% degradation (*T*_10%_) also decrease with an increase in lignin polyol content, possibly due to residual PC and its oligomers in the lignin polyols. In the derivative weight change curve (Figure 9b), the incorporation of lignin polyol significantly reduced the degradation rate of foams, enhancing their thermal stability. This stability can be attributed to lignin's complex aromatic structure and the lignin polyol's strong carbonate linkage. Additionally, the residue content of the foams at 800°C was highest in the lignin‐based foams, ranging from 12% to 15.3%, compared to 9.7% in the control foams. This increased residue content is attributed to lignin's robust and complex structure, which is more resistant to thermal degradation. Foams containing both synthesized lignin polyol and soy polyol (LP30‐SP30) also exhibited high thermal stability. This is evident from the low DTG peak intensity around 370°C and the higher residue content (13.7%), which can be attributed to the presence of lignin.

#### 3.3.5 Biodegradation

The aerobic biodegradation of control foam (made entirely with petrochemical polyols), LP30‐SP30, and LP40 PU foams under composting conditions at 58 ± 2°C and 50 ± 5% relative humidity was assessed. The extent of mineralization was evaluated by carbon dioxide evolution over 120 days.

Biodegradation generally occurs in three main stages. The first stage, the lag phase, is characterized by slow degradation as microbes adapt to the substrate material. This is followed by the active phase, during which the microbial population increases rapidly, leading to the secretion of enzymes that accelerate degradation. Finally, the plateau phase is reached, where microbial activity declines due to the diminishing availability of carbon sources, and biodegradation stabilizes [48]. The type of secreted enzyme depends on the chemical structure of the polymeric substrate and the surrounding environment [49].

In PU degradation, both bacteria and fungi play roles, but fungi are the primary agents [50]. The mode of PU degradation occurs through either hydrolysis or oxidation, depending on the chemical composition of the polyol. Polyester polyol‐based PU primarily undergoes hydrolytic degradation [10], whereas polyether polyol‐based PU degrades through oxidation [51]. Ester linkages are more susceptible to microbial attack than ether linkages [52]. During biodegradation, fungi produce extracellular hydrolytic enzymes such as esterases and proteases, which break down ester and urethane bonds [10]. Additionally, oxidative enzymes such as peroxidases and laccases are responsible for degrading ether bonds [53, 54].

As shown in Figure 9c, lignin‐based PU foams (LP40) and lignin‐soy foams (LP30‐SP30) exhibited higher carbon dioxide evolution than the foams made with 100% petroleum‐based foams (control). The higher biodegradation rate of LP40 (39.8% at 120 days), as presented in Figure 9d, can be attributed to the presence of hydrolyzable carbonate linkages in the synthesized lignin polyol, in contrast to the nonhydrolyzable polyether chains in the petroleum‐based polyol used for the control foam (35.2% at 120 days). However, LP30‐SP30 demonstrated even higher mineralization than LP40. The increased biodegradation of LP30‐SP30 foams (42.6%) can be attributed to the hydrolytic effect of ester linkages in the soft segment of the PU foam due to the presence of soy‐polyol [2].

Ester linkages, predominantly found in soy polyol, are more susceptible to hydrolysis than carbonate linkages due to the generation of carboxylic acids during hydrolysis, which further catalyzes their breakdown [2]. Another factor contributing to the slightly lower mineralization of LP40 relative to LP30‐SP30 is the higher lignin concentration in LP40. While some studies have shown that white‐rot fungi can effectively biodegrade lignin [55, 56, 57], compost environment is not the most favorable degradable environment for lignin. Also, the main lignin degradation product is humic compounds (found mainly in soils) [58], which is not detected in the employed method, which relies on carbon dioxide detection.

Figure 9e highlights the lag phase duration of the developed foams. The results indicate that control foams took ≈40 days for biodegradation to initiate, whereas LP40 and LP30‐SP30 foams began degrading within 25 and 5 days, respectively. This further confirms the susceptibility of ester linkages to biodegradation compared to the carbonate and ether bonds. Additionally, the more extended lag phase of LP40 suggests that its high lignin concentration (9 wt%) may have contributed to slower degradation, owing to lignin's complex aromatic structure and its high functionality, which increases foam's crosslinking density. In the case of the control foam, it was surprising to see any CO_2_ detection, but the presence or production of ester due to oxidation may be responsible for the partial degradation at elevated temperature.

## 4 Conclusions

This study presents the first successful synthesis and integration of lignin‐based polycarbonate polyols into flexible polyurethane foam formulations. Lignin was successfully modified through PC oxyalkylation and DMC transesterification. This resulted in a reactive polyol with favorable hydroxyl values and workable viscosities suitable for high‐performance foam applications. These findings validate the technical feasibility of using lignin as a functional polyol precursor in polycarbonate chemistry. The improved mechanical strength, thermal stability, shock absorption, and biodegradability of the resulting foams not only emphasize lignin's potential as a value‐added biopolymer but also address key industry demands for more environmentally friendly and durable materials. These findings are particularly relevant for automotive seating applications, where stringent performance requirements must be balanced with sustainability goals. Moreover, the demonstrated compatibility with soy polyol opens avenues for hybrid bio‐based formulations with even greater environmental benefits and higher biobased carbon content. Beyond their performance in flexible foam formulations, the lignin‐based polyols developed in this study also offer promising advantages with respect to scalability and sustainability. Technical lignins are widely available as relatively low‐cost byproducts of the pulp and biorefinery industries, providing reliable and renewable feedstock for large‐scale polyol production. The use of lignin not only displaces fossil‐derived carbon but may also improve environmental metrics such as global warming potential and fossil fuel depletion. Furthermore, the method used here occurs under relatively mild conditions, reducing both energy input and processing time. These factors position lignin‐based polyols as credible candidates for industrial adoption, particularly in applications seeking high‐performance, bio‐based, with lower‐carbon footprint materials.

## Supporting Information

Additional supporting information can be found online in the Supporting Information section. Additional supporting information can be found online in the Supporting Information section. **Supporting Fig. S1**: Expansion from HSQC spectra of precipitated propylene carbonate oxyalkylated lignin (OL) from oxyalkylation reaction (a) without PEG and (b) with PEG. **Supporting Fig. 2**: Scanning Electron Microscopy (SEM) of developed control and lignin‐based flexible PU foams at 50x, 100x, and 150x magnification. Lignin‐based foams were formulated using Ligol A. **Supporting Fig. 3**: Thermogravimetric analysis (TGA) thermogram (a) and differential thermogravimetric (DTG) curve (b) of control and lignin polyol (LP)‐based foams (20, 30 and 40 petroleum‐based polyol substitution). **Supporting Fig. 4**: Expansion of a ^31^P{^1^H} NMR spectrum of lignin polyol after propylene carbonate oxyalkylation step. Lignin polyol samples are phosphitylated for ^31^P quantification and analysis. **Supporting Fig. 5**: Aliphatic hydroxyl group region of ^31^P{^1^H} NMR spectra of lignin polyols after propylene carbonate oxyalkylation and dimethyl carbonate transesterification reaction steps. Lignin polyol samples were phosphitylated for ^31^P{^1^H} NMR quantification and analysis. **Supporting Fig. 6**: FTIR‐ATR spectra of synthesized lignin polyols after (a) Propylene carbonate oxyalkylation (b) dimethyl carbonate transesterification reaction. **Supporting Fig. 7**: Expansion of ^1^H NMR spectra of synthesized lignin polyols after propylene carbonate oxyalkylation and dimethyl carbonate transesterification reaction step. **Supporting Fig. 8**: Expansion of ^13^C{^1^H} NMR spectra of synthesized lignin polyols after propylene carbonate oxyalkylation and dimethyl carbonate transesterification reaction step. **Supporting Fig. 9**: Expansion from HSQC spectrum of lignin polyol after dimethyl carbonate (DMC) transesterification reaction. **Supporting Fig. 10**: Expansion from HMBC spectra of synthesized lignin polyols in (a) propylene carbonate oxyalkylation and (b) dimethyl carbonate transesterification reaction. **Supporting Fig. 11**: Test specimen geometry according to ASTM D3574 for (a) tensile and elongation at break (b) compressive force deflection, density, and support factor, and (c) tear strength assessments. **Supporting Fig. 12:** Real‐time acceleration response during an impact test on the foam sample. The peak G‐Value corresponds to the maxium acceleration recorded immidately after the weight was dropped onto the foam. **Supporting Fig. 13**: Designed drop tester for measuring shock absorption of developed PU flexible foams. **Supporting Fig. 14**: Cushion impact test before (left) and after (right) impact using the designed impact tester. The accelerometer and extra weight blocks are installed on the top platen to measure the shock response and increase the weight incrementally, respectively, for generating cushion curves. **Supporting Fig. 15**: Schematic diagram of thermophilic anaerobic biodegradation study of lignin‐based flexible PU foams under compost environment. **Supporting Fig. 16**: In‐house built direct measurement respirometer (DMR) for biodegradation study. Supporting Fig. S17: One‐way ANOVA (P ≤ 0.05) statistical analysis of glass transition temperature (Tg) of precipitated propylene carbonate oxyalkylated lignin (left) and dimethyl carbonate transesterified lignin (TL). **Supporting Table 1**: Measured lignin properties, including lignin source, isolation method, elemental analysis, moisture content, molecular weight, and glass transition temperature. **Supporting Table 2**: Hydroxy moieties of the lignin used in this study, measured by quantitative ^31^P‐NMR spectroscopy. Samples were phosphitylated prior to analysis. **Supporting Table 3**: Physicochemical parameters of compost soil prior to biodegradation test. **Supporting Table 4**: Carbon content analysis of biodegradation test samples measured using CHNS/O Elemental Analyzer. **Supporting Table 5**: Foam reactivity measured according to ASTM D7487.

## Author Contributions

**Enoch** **Kofi Acquah**: conceptualization (equal), data curation (lead), formal analysis (lead), methodology (lead), validation (equal), visualization (equal), writing – original draft (lead), **Daniel Holmes**: data curation (supporting), formal analysis (supporting), methodology (supporting), validation (supporting), visualization (supporting), writing – review and editing (supporting), **Kevin Dunne**: data curation (supporting), formal analysis (supporting), methodology (supporting), visualization (supporting), **Anibal Bher**: data curation (supporting), formal analysis (supporting), methodology (supporting), visualization (supporting), **Saeid Ansari Sadrabadi**: data curation (supporting), formal analysis (supporting), investigation (supporting), methodology (supporting), visualization (supporting), **Amin Joodaky**: funding acquisition (equal), investigation (supporting), methodology (supporting), project administration (equal), resources (equal), supervision (equal), validation (equal), visualization (equal), writing – review and editing (supporting), **Rafael Auras**: conceptualization (equal), data curation (supporting), formal analysis (supporting), funding acquisition (equal), investigation (supporting), methodology (equal), project administration (equal), resources (equal), supervision (equal), validation (supporting), visualization (equal), writing – review and editing (supporting), **Mojgan Nejad**: conceptualization (lead), formal analysis (supporting), funding acquisition (lead), investigation (equal), methodology (equal), project administration (lead), resources (lead), supervision (lead), validation (equal), visualization (supporting), writing – review and editing (lead).

## Funding

This work was supported by United Soybean Board, MTRAC (Grant Case‐48166 of the 21st Century Jobs Trust Fund), and National Institute of Food and Agriculture (McIntire Stennis, 1021850).

## Conflicts of Interest

The authors declare no conflicts of interest.

## Supporting information

Supplementary Material

## Data Availability

The data that support the findings of this study are available from the corresponding author upon reasonable request.
