# Supplementary material for: Synthesis and Characterization of Lignin‐Based Polycarbonate Polyols for Flexible Polyurethane Foam Application
Source: ChemSusChem. 2026 Jan 29;19(2):e202502528. doi: 10.1002/cssc.202502528 (PMC12854248; doi:10.1002/cssc.202502528)
Supplement: Supplementary file 1 — Supplementary Material [file CSSC-19-e202502528-s001.pdf]

Synthesis and Characterization of Lignin-Based Polycarbonate Polyols for Flexible Polyurethane Foam Application

Supplementary Information

Enoch Kofi Acquah,[1] Daniel Holmes,[3] Kevin Dunne,[1] Anibal Bher,[4] Saeid Ansari Sadrabadi,[4] Amin Joodaky,[4] Rafael Auras,[4] and Mojgan Nejad[1,2 *]

[1] Chemical Engineering and Materials Science Department, Michigan State University, 428 S. Shaw Ln Rm 2100, East Lansing, MI 48824, USA, acquahen@msu.edu, dunnekev@msu.edu

[2] Department of Forestry, Michigan State University, 480 Wilson Rd, East Lansing, MI 48824, USA, nejad@msu.edu

[3] Department of Chemistry, Michigan State University, 578 S. Shaw Ln, East Lansing, MI 48824, holmesd5@msu.edu

[4] School of Packaging, Michigan State University, 448 Wilson Rd, East Lansing, MI 48824, ansari10@msu.edu, joodakya@msu.edu, aurasraf@msu.edu

Supplementary Data:

Lignin Properties

Table S1 Measured lignin properties, including lignin source, isolation method, elemental analysis, moisture content, molecular weight, and glass transition temperature.

**Property**	**Result**
Isolation Method	Hydrolysis
Source	Hardwood
Ash Content (wt.%)	2.3
Moisture Content (wt. %)	1.2 ± 0.1
Na (wt %)	0.56
Sulfur content (%)	0.28
M_n (Da)	1110
M_w (Da)	5480
Dispersity	4.9
Tg (°C)	156

Table S2: Hydroxy moieties of the lignin used in this study, measured by quantitative ^{31}P-NMR spectroscopy. Samples were phosphitylated prior to analysis.

**Hydroxy Moiety Content (mmol/g)**	**Result**
Aliphatic	2.66
Condensed Phenolic	0.43
Syringyl	1.46
Guaiacyl	0.48
Hydroxyphenyl	0.12
Carboxylic Acid	0.28
Total Hydroxy Content	5.43

Structural Analysis of Precipitated Oxyalkylated Lignin

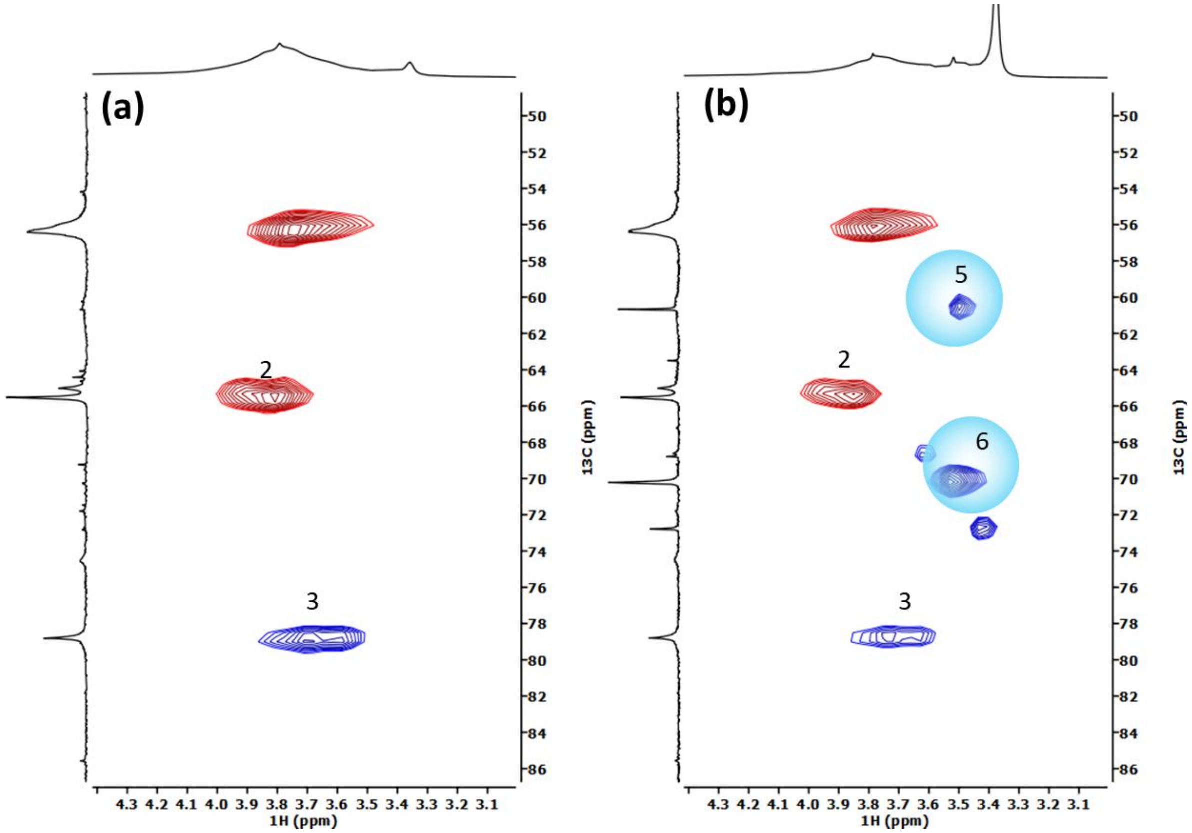

Figure S1: Expansion from HSQC spectra of precipitated propylene carbonate oxyalkylated lignin (OL) from oxyalkylation reaction (a) without PEG and (b) with PEG.

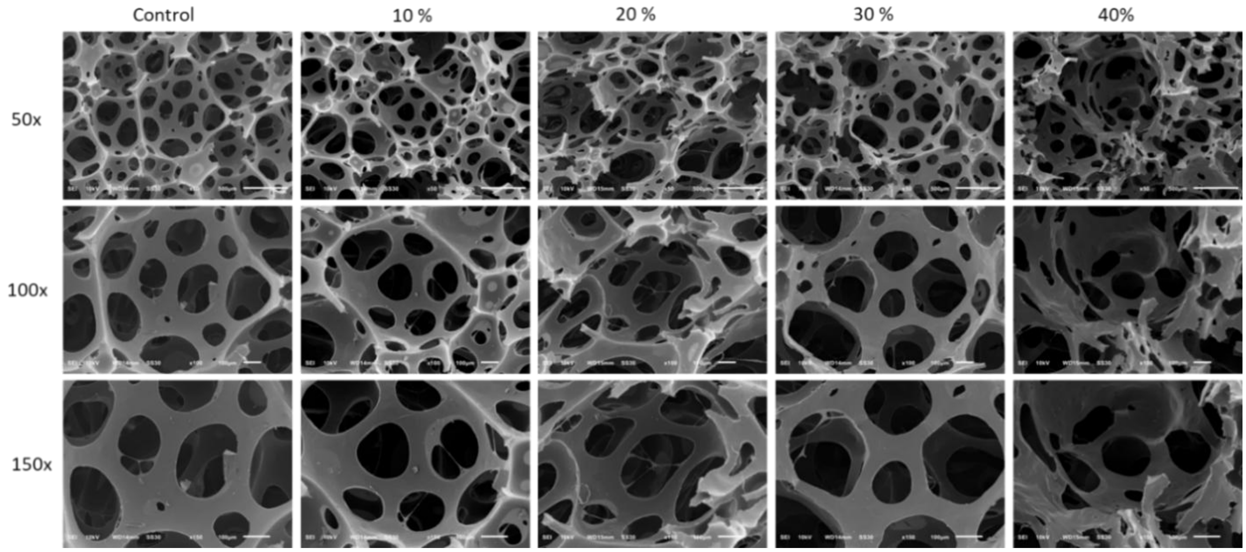

Figure S2: Scanning Electron Microscopy (SEM) of developed control and lignin-based flexible PU foams at 50x, 100x, and 150x magnification. Lignin-based foams were formulated using Ligol A.

Thermogravimetric Analysis (TGA)

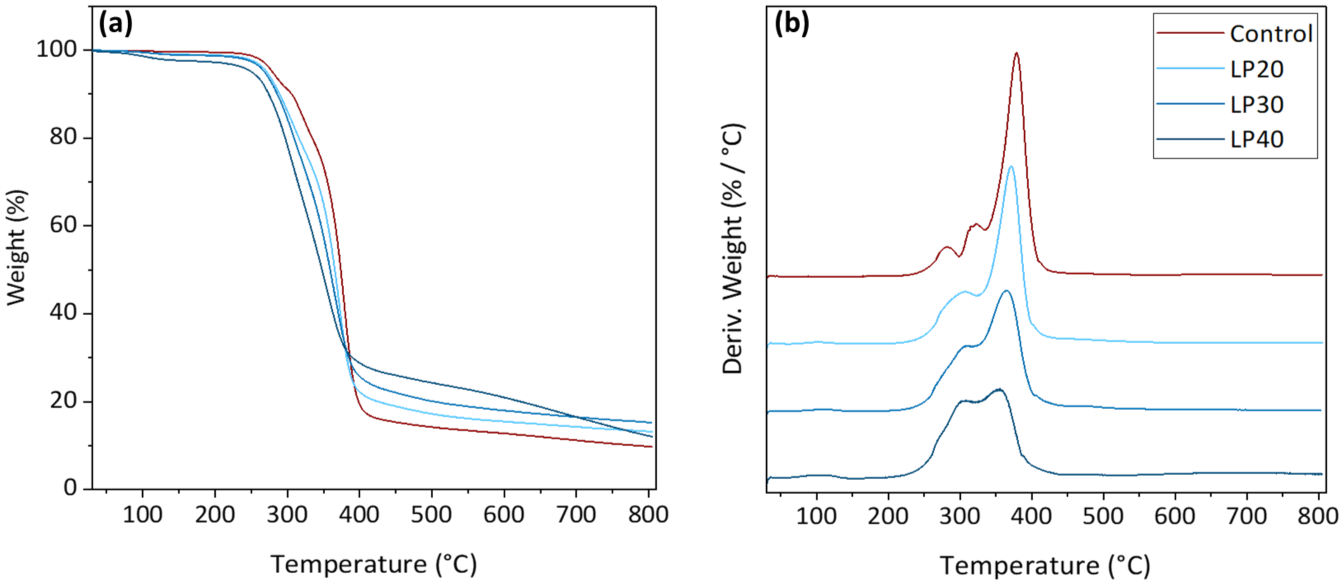

Figure S3: Thermogravimetric analysis (TGA) thermogram (a) and differential thermogravimetric (DTG) curve (b) of control and lignin polyol (LP)-based foams (20, 30 and 40 petroleum-based polyol substitution).

Structural Analysis of Crude (Unprecipitated) Oxyalkylation and Transesterification Lignin Polyol

The propylene carbonate (PC) oxyalkylation was an important step for two reasons. First, it converts sterically hindered phenolic OH functionalities to aliphatic hydroxyl groups, improving lignin's overall reactivity towards isocyanate. Second, to facilitate its subsequent reaction towards dimethyl carbonate (DMC) to produce polycarbonate polyols. It is also important to note that carboxylic hydroxyl groups of lignin also react with propylene carbonate to produce aliphatic OH group terminated derivatives.[1] PC, being a liquid at room temperature, was preferred over ethylene carbonate as the cyclic alkyl carbonate of choice because it yields a polyol mixture with significantly lower viscosity, a crucial factor for foam manufacturing.

^{31}P NMR analysis was performed on the crude lignin polyol (unprecipitated), after phosphitylating, to confirm the success of the oxyalkylation reaction. The absence of both phenolic (137.3 -144.6 ppm) and carboxylic hydroxyl (134 – 135.9 ppm) groups in lignin was evident (**Figure S4**), indicating the conversion of these functional groups to aliphatic hydroxyl groups. During the oxyalkylation of lignin, deprotonated phenolic and carboxylic hydroxyl groups undergo a nucleophilic attack on the alkyl carbon of propylene carbonate, resulting in the generation of carbon dioxide gas.[1–4] The peaks in the range of 147.2-146.86 ppm in ^{31}P{^{1}H} NMR spectra were identified as derived from PEG's aliphatic hydroxyl groups. Furthermore, the emergence of two distinct peaks at 147.3 and 145.6 ppm suggests the possible formation of propylene glycol (PG) or oligomeric glycols, likely originating from PC-ring opening from residual water molecules or transesterification side reactions.[58] Thus, the oxyalkylation product mainly contains modified lignin, PEG, and oligomeric glycols. The quantitative analysis of the total hydroxyl content of the mixture yielded a value of 6.51 mmol/g (equivalent to 365 mg

KOH/g). This value falls beyond the acceptable range of hydroxyl values (28 to 180 mg KOH/g) for polyols intended for use in flexible polyurethane applications.

After the oxyalkylation step, transesterification with dimethyl carbonate (DMC) was carried out to produce polycarbonate polyols, generating methanol as a byproduct. The $^{31}P\{^{1}H\}$ NMR analysis of the transesterification product, as shown in **Figure S5**, indicates a significant reduction in peaks corresponding to PG at 147.3 and 145.6 ppm. Similarly, the intensity of the peak associated with the aliphatic hydroxyl group of PEG (147.2–146.86 ppm) decreased. In contrast, the aliphatic hydroxyl group of lignin showed a relatively lower reduction, likely due to its bulky structure and the presence of secondary hydroxyl groups formed during oxyalkylation with propylene carbonate. These structural factors may have contributed to lignin's lower reactivity toward DMC. Secondary hydroxyl groups tend to exhibit lower reactivity towards dimethyl carbonate compared to primary aliphatic hydroxyl groups,[5] which are predominantly present in PEG.

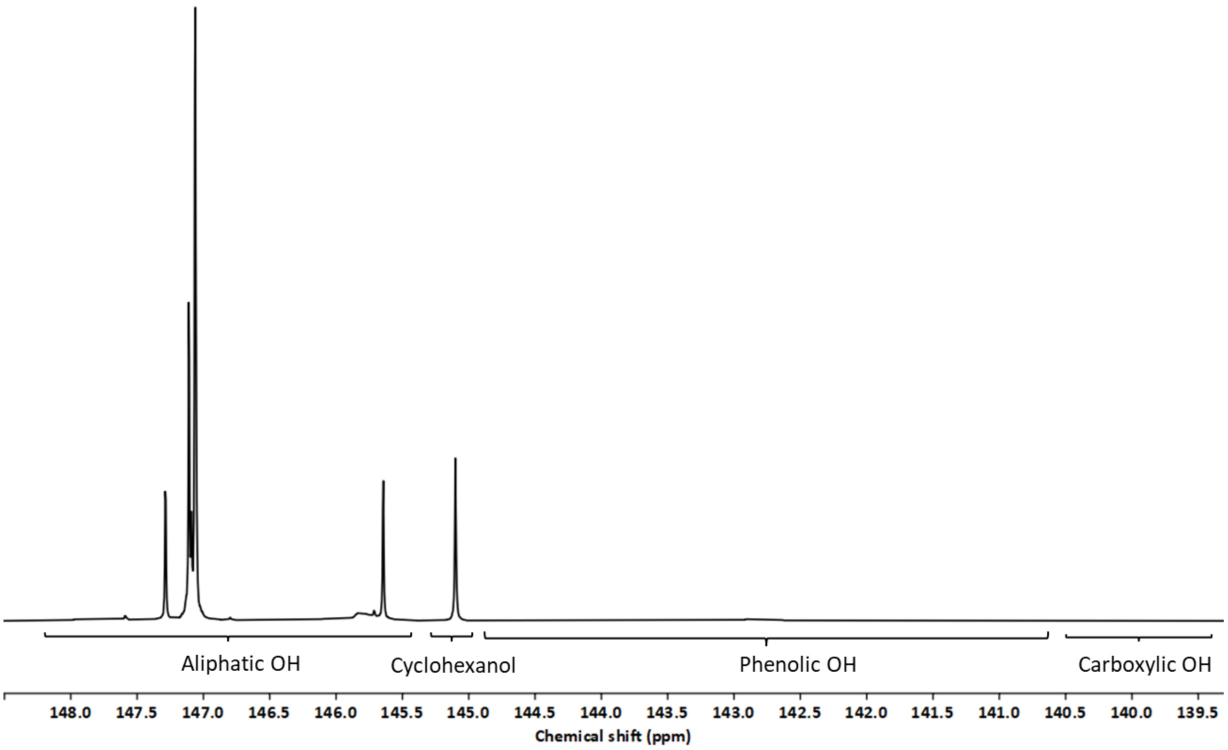

Figure S4: Expansion of a $^{31}P\{^{1}H\}$ NMR spectrum of lignin polyol after propylene carbonate oxyalkylation step. Lignin polyol samples are phosphitylated for ^{31}P quantification and analysis.

Quantitative analysis of polyethylene glycol and oligomeric glycols aliphatic OH group reduction, obtained from integrating their respective $^{31}P\{^{1}H\}$ NMR peaks, after 30 minutes of transesterification was 43.95 and 91.5%, respectively. This further demonstrates that primary hydroxyl groups are more reactive towards dimethyl carbonate. The total hydroxyl content of the reaction mixture after the transesterification reaction yielded a value of 3.19 mmol/g (179 mg KOH/g), making it more suitable for use in flexible PU formulation.

The FTIR spectrum of the transesterification reaction product indicates an increase in the ether linkage at 1260 cm^{-1} and the emergence of a new carbonyl (C=O) peak at 1740 cm^{-1} as seen in **Figure S6**. This new peak is associated with the carbonate linkage formed during the

transesterification reaction with dimethyl carbonate. These carbonate linkages have been reported to improve mechanical properties and resistance to hydrolysis and oxidation of foams.[6–12] The decrease in the OH peak at 3433 cm^{-1} is consistent with the findings observed in the $^{31}P\{^{1}H\}$ NMR analysis. In total, there was a 51% reduction in the hydroxyl groups of oxyalkylation derivatives after the transesterification reaction.

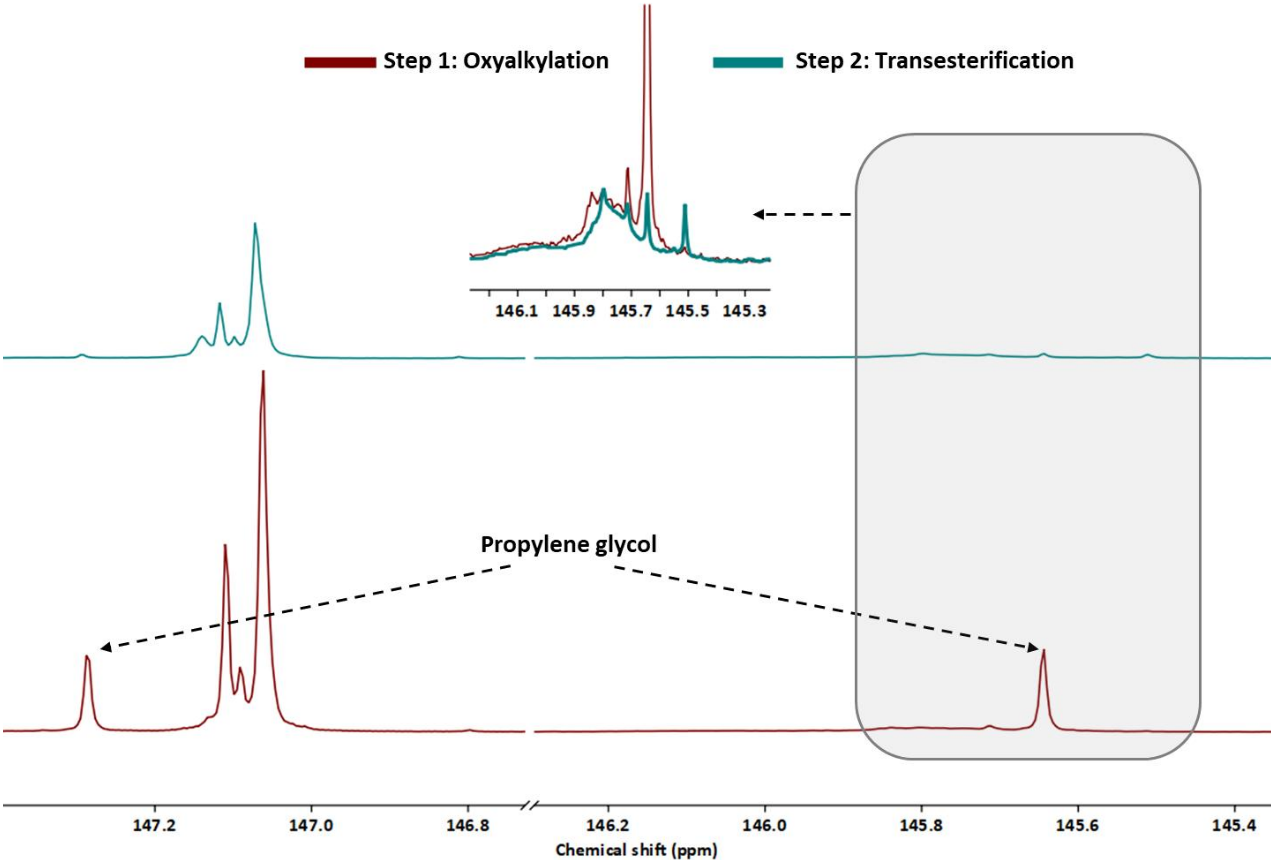

Figure S5: Aliphatic hydroxyl group region of $^{31}P\{^{1}H\}$ NMR spectra of lignin polyols after propylene carbonate oxyalkylation and dimethyl carbonate transesterification reaction steps. Lignin polyol samples were phosphitylated for $^{31}P\{^{1}H\}$ NMR quantification and analysis.

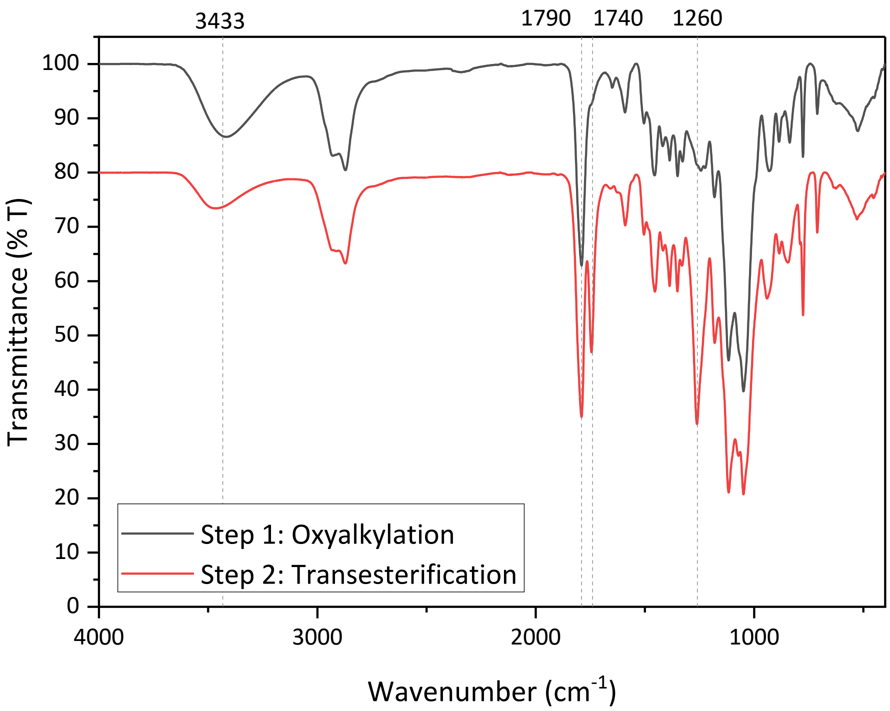

Figure S6: FTIR-ATR spectra of synthesized lignin polyols after (a) Propylene carbonate oxyalkylation (b) dimethyl carbonate transesterification reaction.

^{1}H NMR analysis of the oxyalkylation product displays signals within the 1-1.5 ppm range, as seen in **Figure S7**, which can be attributed to the newly formed methyl group resulting from the reaction of lignin with propylene carbonate.[13] The doublet at 0.98 ppm is associated with the methyl group on propylene glycol generated through side reactions. Further analysis of the transesterification reaction product revealed a decrease in the methyl proton at 0.98 ppm which corresponds to propylene glycol side product. ^{1}H chemical shift signals at 3.41, 3.48 and 3.485 ppm can be attributed to methylene protons in PEG. The reduction in the proton signal intensity at 3.485 ppm, corresponding to PEG's characteristic terminal methylene protons, is consistent with the results from ^{31}P{^{1}H} NMR and FTIR. This can be attributed to the transesterification

reaction between PEG hydroxyl groups and DMC, leading to a decrease in its hydroxyl signals. Subsequently, two new peaks emerged at 3.62, and 4.18 ppm.

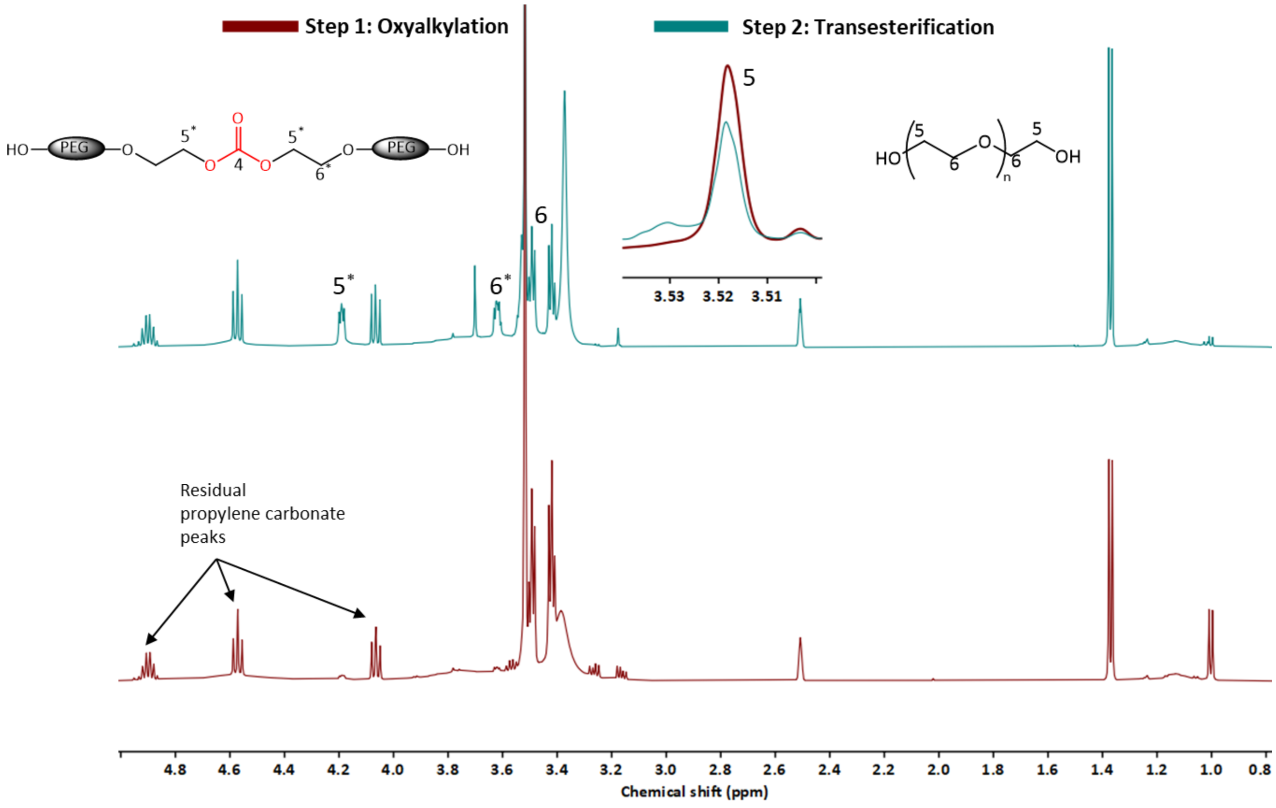

Figure S7: Expansion of ^{1}H NMR spectra of synthesized lignin polyols after propylene carbonate oxyalkylation and dimethyl carbonate transesterification reaction step.

HSQC analysis of these new proton signals confirmed that they are methylene (CH_2) protons as seen in **Figure S9**. The appearance of these protons suggests the potential for random co-polymerization between lignin and polyethylene glycol during the transesterification step. Additionally, a new methyl proton signal was observed at 3.7 ppm. This signal appears as a

singlet, which may have originated from either residual DMC or a terminal methyl carbonate group on a PEG molecule.

The confirmation of the formation of polyethylene glycol adducts (resulting from the reaction of PEG with propylene carbonate) was conducted using $^{13}C\{^1H\}$ NMR. In addition to the carbonyl carbon peak (155.4 ppm) of PC, a new minor peak at 155.07 ppm emerged as seen in **Figure S8**, indicating the presence of a PEG adduct in the oxyalkylation products. The intensity of this peak suggests that only a small amount of this product was present. The carbonyl carbon signal at 155.07 ppm increased significantly by the end of the transesterification reaction. This is due to the reaction between PEG hydroxyl groups and DMC leading to the introduction of carbonate linkages. $^{13}C\{^1H\}$ NMR spectra also show the emergence of two methylene carbon peaks at 67.46 and 68.97 ppm, thus confirming the reaction of PEG with dimethyl carbonate. Additionally, a new carbonyl carbon emerged at 155.7 ppm.

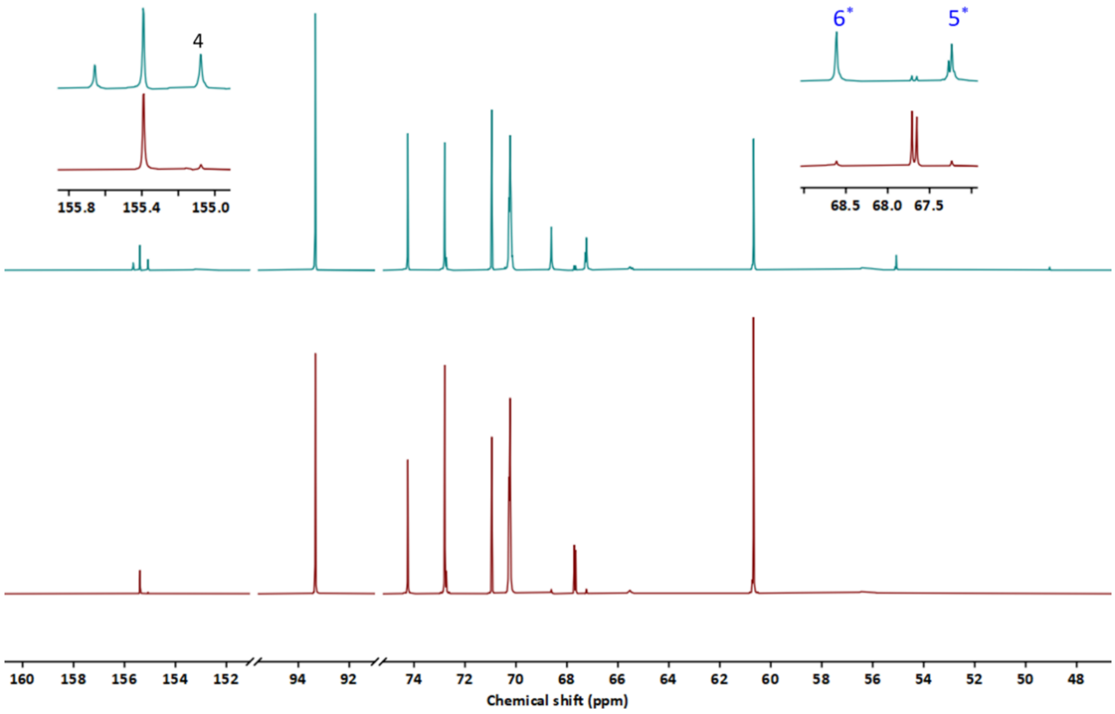

Figure S8: Expansion of $^{13}C\{^{1}H\}$ NMR spectra of synthesized lignin polyols after propylene carbonate oxyalkylation and dimethyl carbonate transesterification reaction step.

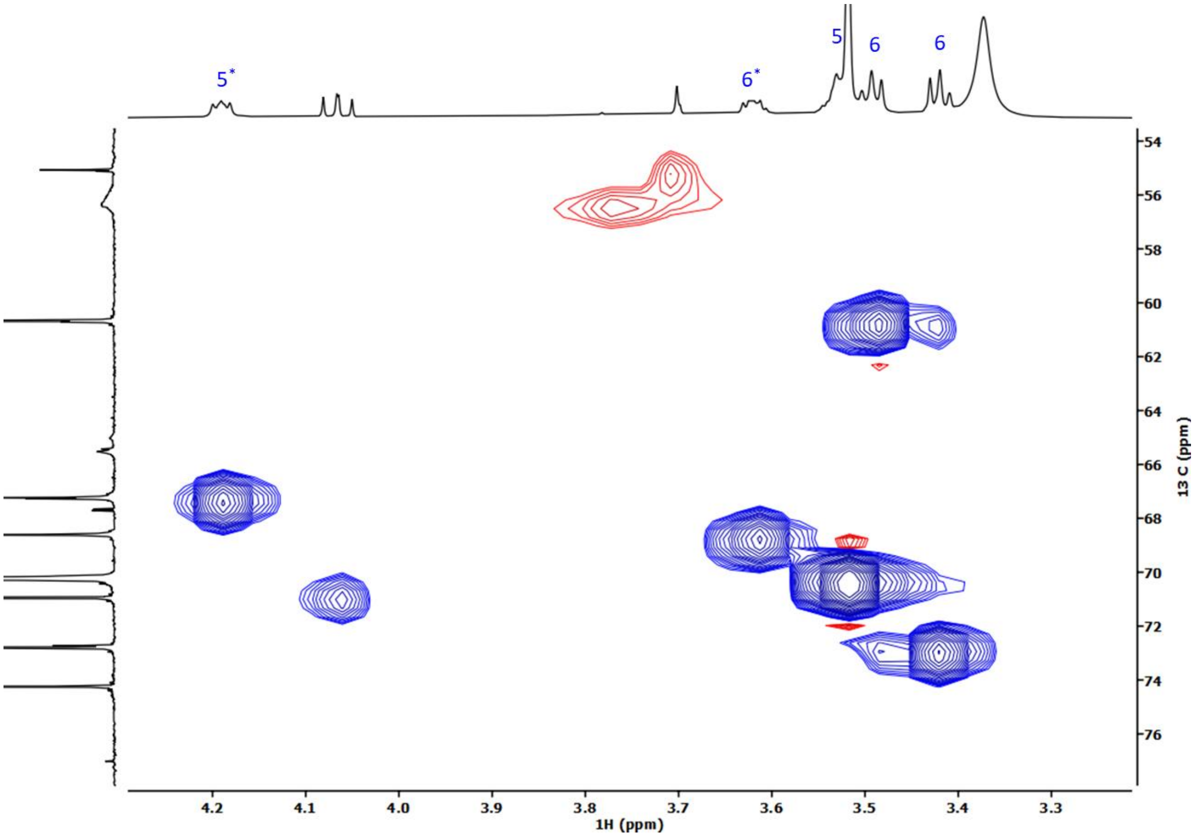

Figure S9: Expansion from HSQC spectrum of lignin polyol after dimethyl carbonate (DMC) transesterification reaction.

To elucidate the structure of the polyol repeating unit predominantly formed after the transesterification reaction, HMBC was conducted. The HMBC spectra, presented in **Figure S10**, shows that the methylene proton at 4.18 ppm has a cross-peak with a carbonyl carbon at 155.07. Methylene carbon at 68.97 ppm also correlates with the proton signal at 4.18 ppm, thus confirming the presence of the PEG-PEG repeating unit in the polymer chain. The carbonyl carbon at 155.7 has a cross-peak with both methylene protons at 4.18 ppm and methyl proton at 3.7 ppm. This cross-peak confirms the presence of a terminal methyl carbonate group on some

PEG molecules. The presence of this side product was unexpected considering only 0.5 equivalent of DMC was used in the transesterification reaction indicating the need to extend the reaction time longer to enable further chain extension. Overall, there was no clear evidence from the NMR analysis to confirm the formation of lignin-PEG and lignin-lignin repeating units. Therefore, an in-depth NMR analysis of precipitated modified lignin (oxyalkylated lignin (OL) and transesterified lignin (TL)) in both reaction steps was performed to further validate the presence of these units.

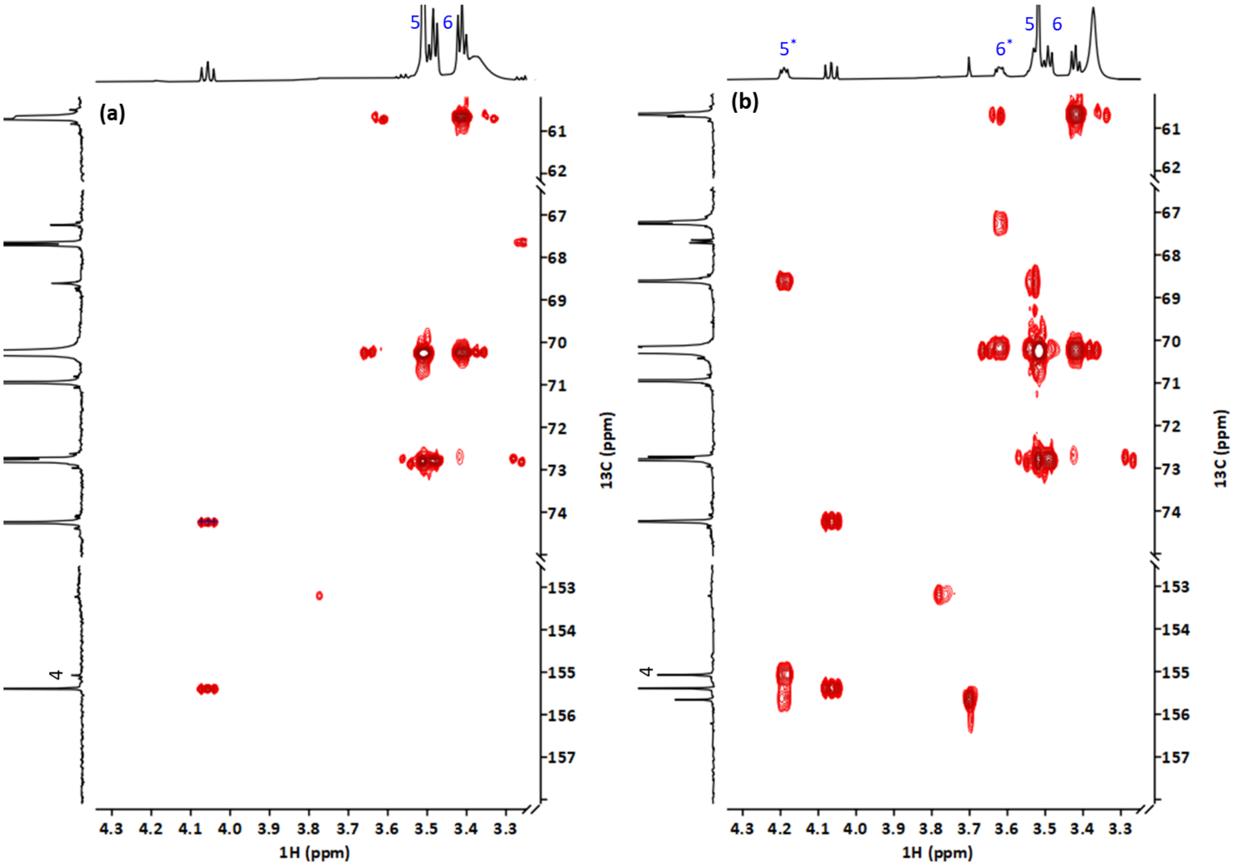

Figure S10: Expansion from HMBC spectra of synthesized lignin polyols in (a) propylene carbonate oxyalkylation and (b) dimethyl carbonate transesterification reaction.

Detailed Methodology:

Lignin Characterization

Various analytical techniques were employed to characterize lignin and assess its composition and properties. The ash content was measured following the TAPPI T 212 om-93 standard method, where lignin samples were heated at 525 °C for 4 hours, and the residual mass was recorded. Elemental composition was analyzed using Inductively Coupled Plasma Atomic Emission Spectroscopy (ICP-AES). The hydroxyl group content was determined via Phosphorus-31 Nuclear Magnetic Resonance (^{31}P NMR) spectroscopy, following established protocols. [14–17] Molecular weight distribution was evaluated using gel permeation chromatography (GPC), with prior acetylation to improve solubility in tetrahydrofuran (THF). All analyses were performed in triplicate, and the average values were reported.

Polyol Characterization

Hydroxyl Value (OHV)

The hydroxy contents of the polyol were measured using ^{31}P-NMR spectroscopy, using a method similar to that used for lignin characterization. The hydroxyl value (OHV), measured in mg KOH/g, of the polyol was computed by multiplying the total hydroxyl content (mmol/g) by 56.1.

Viscosity

The lignin viscosity of developed polyols was assessed at ambient temperature employing a Discovery HR-1 hybrid rheometer manufactured by TA Instruments. A 40 mm stainless steel Peltier plate geometry with a 1 mm gap was utilized, with samples sandwiched between the

plates and trimmed before analysis. Viscosity measurements were conducted under a constant shear rate of 50 s^{-1}, with the recorded viscosity representing the average value over 60 seconds.

Modified lignin content

The quantification of modified lignin content in the polyol at the end of PC oxyalkylation reaction was conducted gravimetrically. Initially, the mass of a dry 50 mL beaker and a ceramic filtration device, with a pore size of 10-15 µm, was determined. Subsequently, 1 g of each polyol was combined in a beaker with 20 mL of acidified water (pH 2) and vigorously agitated until the modified lignin precipitated entirely. Following precipitation, the mixture was subjected to vacuum filtration using ceramic filters. The resulting samples were left to dry overnight in a vacuum oven at 85 °C and subsequently cooled in a desiccator. The mass of the dry-modified lignin was computed by subtracting the mass of the dry ceramic filter from the combined dry mass of the lignin and filter. Consequently, the modified lignin content was calculated using equation (1).

$$Modified\ lignin\ content\ (\%) = \frac{modified\ lignin\ mass}{lignin\ polyol\ mass} \times 100 \quad [1]$$

Chemical Structural Analysis

The oxyalkylation and transesterification reaction were confirmed using FTIR, ^{1}H, ^{13}C, HSQC, and HMBC NMR experiments. Samples for NMR analysis were prepared using the following protocol: 50 mg of 1,3,5 trioxane was dissolved in 1 mL of the solvent (deuterated dimethyl sulfoxide (DMSO-d_6)) to prepare an internal standard solution. Approximately 40 mg of the synthesized lignin polyol was dissolved in 0.6 mL of the solvent, and the mixture was mixed for 30s in a vortex at 3000 rpm. Finally, 100 µL of the internal standard solution was then added,

and vortexed for 30s at 3000 rpm to produce a uniform solution. 650 µL of the final mixture was then transferred to a 5mm Wilmad NMR tube. The NMR solution was analyzed in an Agilent DDR2 500 MHz NMR spectrometer equipped with 7600AS autosampler, running VnmrJ 3.2 A. For lignin polyol samples, the following parameters were used: ^{1}H NMR (1s relaxation delay and 16 scans), ^{13}C NMR (2 s relaxation delay and 5000 scans), HSQC (1.5 s relaxation delay and 8 scans), and HMBC (2 s relaxation delay and 16 scans). For precipitated lignin samples, the following parameters were used: ^{1}H NMR (4 s relaxation delay and 128 s scans), ^{13}C NMR (2 s relaxation delay and 8000 scans), HSQC (1.5 s relaxation delay and 64 scans), and HMBC (2 s relaxation delay and 40 scans). The infrared spectra of synthesized lignin polyols were measured with a PerkinElmer Spectrum Two FT-IR Spectrometer, in Attenuated Total Reflectance (ATR) mode. Few drops of lignin polyols were placed on the ATR crystal and data was collected using 32 scans. Spectrum was processed with a baseline correction and noise-reduction algorithm after data collection.

Glass Transition Temperature (Tg)

Differential Scanning Calorimetry (DSC) of precipitated lignin samples were conducted using a standard aluminum pan and lid. Approximately 8 mg of each sample was weighed and analyzed using a TA DSC instrument under nitrogen. The testing protocol involved equilibrating the sample at 25°C, ramping the temperature at 20 °C/min to 200 °C, cooling at 20 °C/min back to 25 °C, holding isothermal for 10 minutes, and then ramping again at 20°C/min to 230°C.

Foam Formulation

Foams with varying amounts of lignin polyol were formulated for characterization. 10, 20, 30, and 40 part per hundred polyols (pphp) of the petroleum-based polyols were replaced with the lignin polyol to synthesize flexible polyurethane foams. Foams containing both synthesized

lignin polyol and soy polyol were also formulated to increase the biobased content. The Isocyanate index was set at 100 for all foams. In a 12-ounce paper cup, 100 g of polyol blend, water (3 pphp), gelation catalyst (0.65 pphp), blow catalyst (0.32 pphp), and surfactants (1.5 pphp) were mixed at 3000 rpm using a high-speed mixer for two minutes. Finally, the calculated amount of isocyanate was added using a disposable plastic syringe and mixed at 3000 rpm for 3-5 seconds. The mixture was then quickly poured into a silicone mold conditioned at 65 °C and left to cure at room temperature for about 30 minutes before demolding. After removing the foams from the mold, they were left to post-cure for 2 days at room temperature. Samples were cut into various sizes required for mechanical testing **(Figure S11)** devoid of skin.

Foam Characterization

Mechanical Property Test

The foam samples were cut into mechanical testing specimens without any skin present. These samples were then subjected to property characterization using an Instron Universal Testing machine based on the ASTM D3574 test standard [18] to assess their physical and mechanical properties, including density, compression force deflection (CFD), support factor, tensile strength, ultimate elongation at break, and tear strength. The values obtained were then compared to the control foams made without lignin polyol and the Original Equipment Manufacturer (OEM) standard requirements for automotive seating applications. Six replicates of each foam sample were tested, and the average and standard deviations were reported.

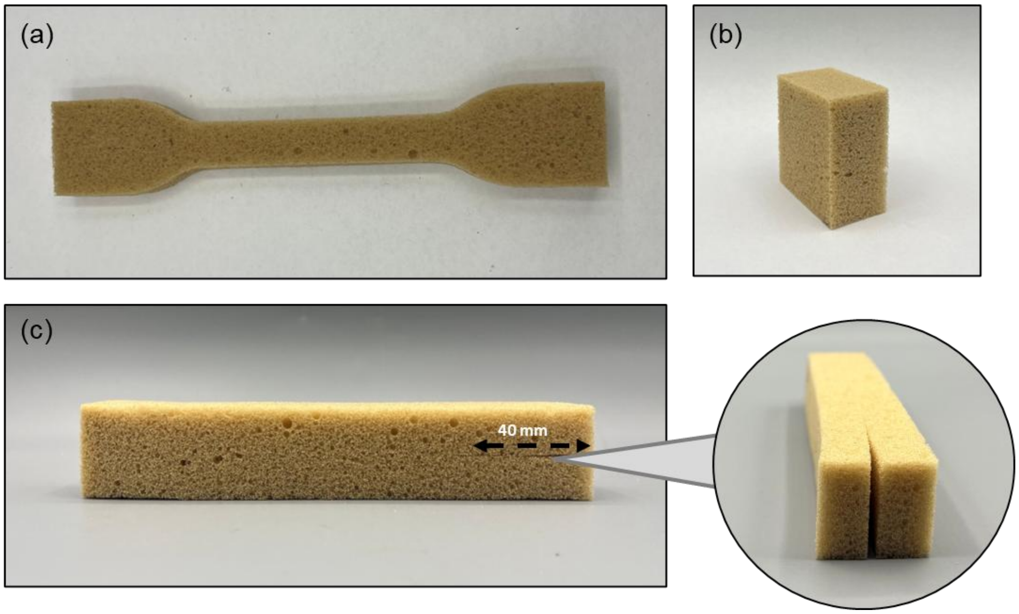

Figure S11: Test specimen geometry according to ASTM D3574 for (a) tensile and elongation at break (b) compressive force deflection, density, and support factor, and (c) tear strength assessments.

Scanning Electron Microscopy

The morphology of foam samples was investigated using scanning electron microscopy (SEM). Samples were cut perpendicular to the foam rise direction. Then, the samples were mounted using carbon tape and made conductive by sputter coating with gold nanoparticles in an argon atmosphere. Images were collected on a JEOL 6610LV SEM with an accelerating voltage of 10 kV and a spot size set to 30. Images were collected at 50x, 100x, and 1000x magnification. Open cell diameters of foams were then measured using ImageJ. The cell diameter of several positions on the foam was determined and the average reported.

Impact Test

The dynamic characteristics of foams are essential information in comfort designs and shock absorption of automobile seats. Cushion curves show the deceleration level in g's (e.g. 9.81 m/s^2)

versus static stresses. A drop test machine was used to perform the required drop tests for generating cushion curves according to ASTM D1596-14. Foam samples measuring 2 by 4 inches with a thickness of 1 inch, giving a top area of 8 square inches, were used. An accelerometer attached to the drop platen was used to record the shock pulse during each drop scenario. The static stress, σ_s, for this impact event was expressed as equation (2):

$$\sigma_s = \frac{W}{A} \quad [2]$$

where W is the weight of the platen, and A is the area. Then, the weight of the platen was increased from 3.5 lbs to 5 lbs in 0.5 lb increments, and each weight was dropped from a height of 6 inches. Since the samples were relatively small, a 6-inch height was opted to avoid excessively fast impacts. The peaks of these pulses were connected to generate a cushion curve, which represents the shock absorption capacity of the foam. Typically, a new sample is used for each test, and tests are repeated five times according to ASTM D1596. This would require a large number of samples, especially when varying both weight and height. However, due to the limited number of samples, foams were reused multiple times.

Impact Tester Design

The platens of the drop tester model (Lansmont Model 23) were too heavy for testing small foam samples. A smaller drop tester was designed using machine design principles and techniques. Several design drawings were created using SolidWorks software and were analyzed for optimization. The natural frequency of the platen was estimated using Finite Element Method software, ABAQUS, to ensure the platen remains relatively flat during the impact. Two flanges with internal ball bearings were used to facilitate the drop and minimize friction between the parts. **Figures S13 and S14** show the designed drop tester used in this study.

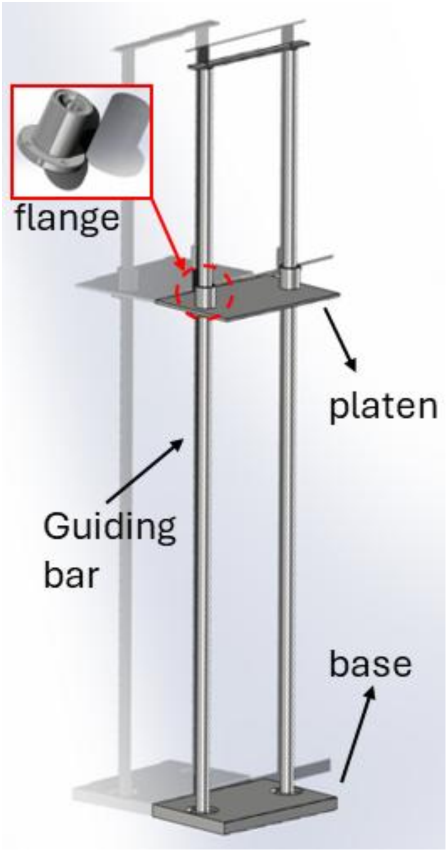

Figure S13: Designed drop tester for measuring shock absorption of developed PU flexible foams

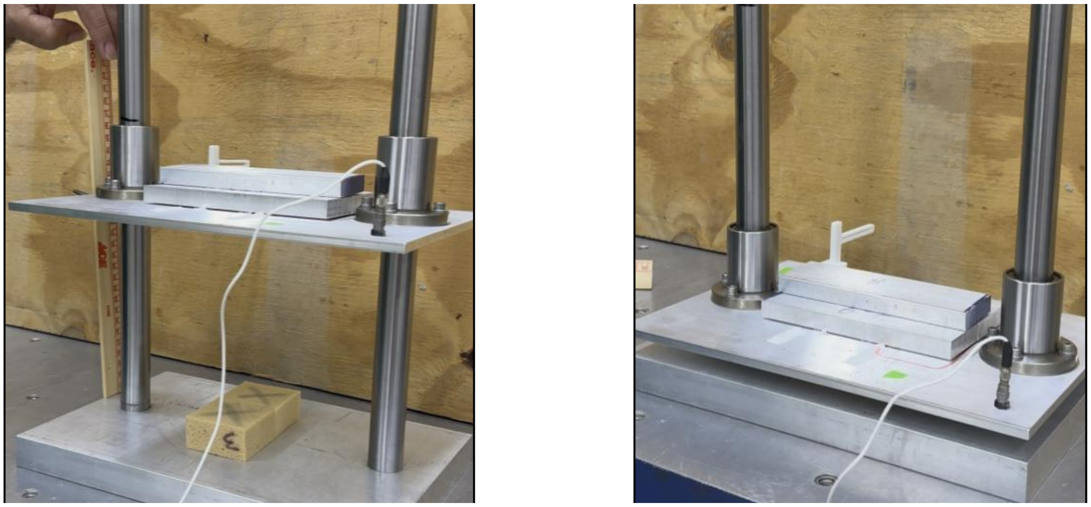

Figure S14: Cushion impact test before (left) and after (right) impact using the designed impact tester. The accelerometer and extra weight blocks are installed on the top platen to measure the shock response and increase the weight incrementally, respectively, for generating cushion curves.

Limitations

Due to limited resources in foam creation, the recommended specifications in ASTM D1596 (e.g., a minimum top foam area of 4 by 4 inches) were not fully adhered to. Additional testing with unused samples is necessary, including trials at varying drop heights, to accurately assess the full shock-absorption capacity of the developed lignin-based foams.

Biodegradation Study

The aerobic biodegradation of control, LP40, and LP30-SP30 foams was evaluated in a compost under controlled composting conditions (58 ± 2 ºC and 50 ± 5% relative humidity (RH)) by analysis of evolved CO_2 using an in-house built direct measurement respirometer (DMR),

equipped with a non-dispersive infrared gas analyzer (NDIR) following ASTM and ISO standards. [19–22]

Compost (manure straw) was acquired from the Michigan State University (MSU) Composting Facility (East Lansing, MI, U.S.), and its physiochemical properties are summarized in **Table S3** (see SI). The compost was sieved through a 10-mm screen to remove inert and large materials and conditioned at approximately 58 ºC until use. Deionized water was added to increase the moisture content of the compost (up to 50%). Additionally, the physiochemical parameters (see Table S4 in Supplementary Information) of the compost were determined.

Each bioreactor (1.9 L) was filled with 400 g of compost, then 8 g of the foam sample, cut into small equal pieces, was added to each bioreactor for testing. To maintain optimal conditions for the biodegradation process, deionized water was injected into each bioreactor twice a week. After each water injection, the bioreactor was shaken to distribute the water and avoid clustering. During the operation of the test, air at 50 ± 5% RH flowed to each bioreactor, and the CO_2 evolved was collected and measured by the near-infrared sensor at regular intervals. The CO_2 evolved from the blank bioreactor (without foam) was considered as the background signal. This value was subtracted from the amount of CO_2 produced by each sample bioreactor to calculate the biodegradation of each sample, where the % biodegradation is the total amount of carbon molecules converted to CO_2. It is calculated according to the equation (3) below. [23]

$$\%\, Mineralization = \frac{(CO_2)_t - (CO_2)_b}{M_t \times C_t \times \frac{44}{12}} \times 100 \qquad [3]$$

where the numerator is the difference between the average of the three bioreactors' cumulative mass of CO_2 evolved for the sample $(\mathrm{CO_2})_\mathrm{t}$, and the average CO_2 evolved from the three blank bioreactors $(\mathrm{CO_2})_\mathrm{b}$. The denominator represents the theoretical amount of CO_2 able to be

produced by the sample. M_t is the total mass of the sample, C_t is the proportion of carbon present in that sample as determined by CHNS/O Analyzer, and 44 and 12 are the molecular mass of CO_2 and the atomic mass of carbon, respectively. **Figures S15** and **S16** (see SI) present an illustration of the biodegradation study.

Elemental Analysis

The carbon content of the different test materials was determined by elemental analysis using a PerkinElmer 2400 Series II CHNS/O Elemental Analyzer (Shelton, CT, USA). The test used about 2 milligrams of each sample weighed in small capsules. A blank, and standard values to establish the k-factors were measured before assessing the samples. Results of elemental analysis can be found in Supplementary Information **(Table S3** in SI**)**.

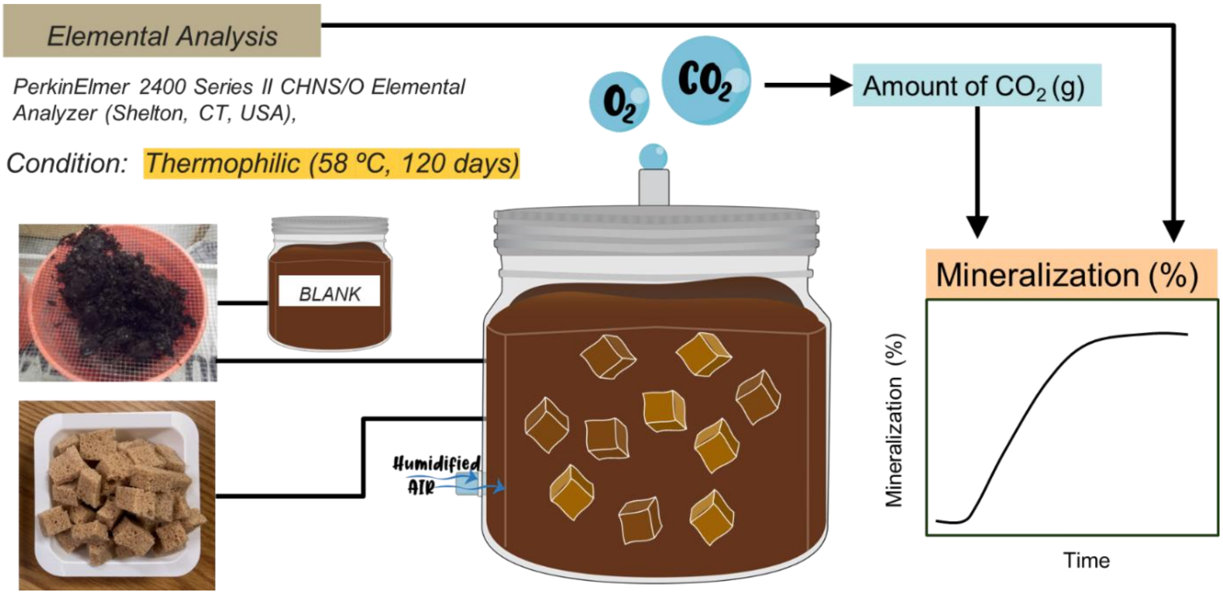

Figure S15: Schematic diagram of thermophilic anaerobic biodegradation study of lignin-based flexible PU foams under compost environment.

Table S3: Physicochemical parameters of compost soil prior to biodegradation test

Tests	Units	Compost
pH	-	7.22
E. C. - Saturation Paste	mmho/cm	13.8
Total Dry Solid	%	55.7
Total Volatile Solid	%	44.4
C/N Ratio		10.3
Total Nitrogen (N)	%	2.27
Total Phosphorus (P)	%	1.36
Total Potassium (K)	%	1.46
Total Calcium (Ca)	%	6.11
Total Magnesium (Mg)	%	1.57
Total Zinc (Zn)	ppm	380
Total Iron (Fe)	ppm	7033
Total Manganese (Mn)	ppm	294
Total Copper (Cu)	ppm	126
Total Carbon (C)	%	23.3
Total Sodium (Na)	%	0.347
Total Aluminum (Al)	%	0.198
Total Sulfur (S)	%	0.505
Total Boron (B)	ppm	39

Table S4: Carbon content analysis of biodegradation test samples measured using CHNS/O Elemental Analyzer

Sample	% Carbon (average)
Cellulose	42.5
Control	65.7
Lignin polyol	54.1
Pure lignin	63.2
LP30-SP30	67.1
LP40	64.0

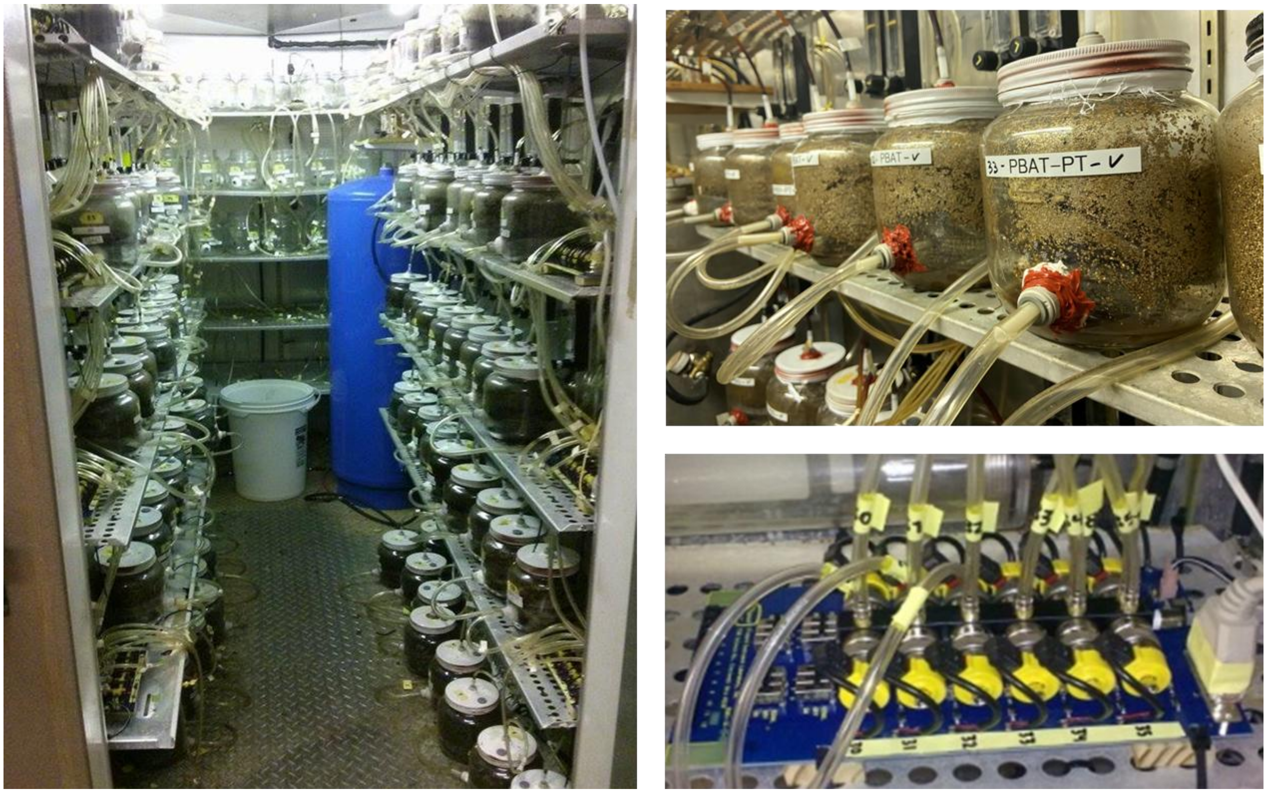

Figure S16: In-house built direct measurement respirometer (DMR) for biodegradation study

Table S5: Foam reactivity measured according to ASTM D7487

Sample	cream time (s)	gel string time (s)	Rise time (s)	Tack free time (s)
Control	6	35	51	150
LP 10	6	30	42	136
LP 20	6	26	34	112
LP 30	6	25	32	75
LP 40	6	22	30	62
LP20-SP20	6	28	37	115
LP30-SP30	6	26	33	77

Statistical Analysis

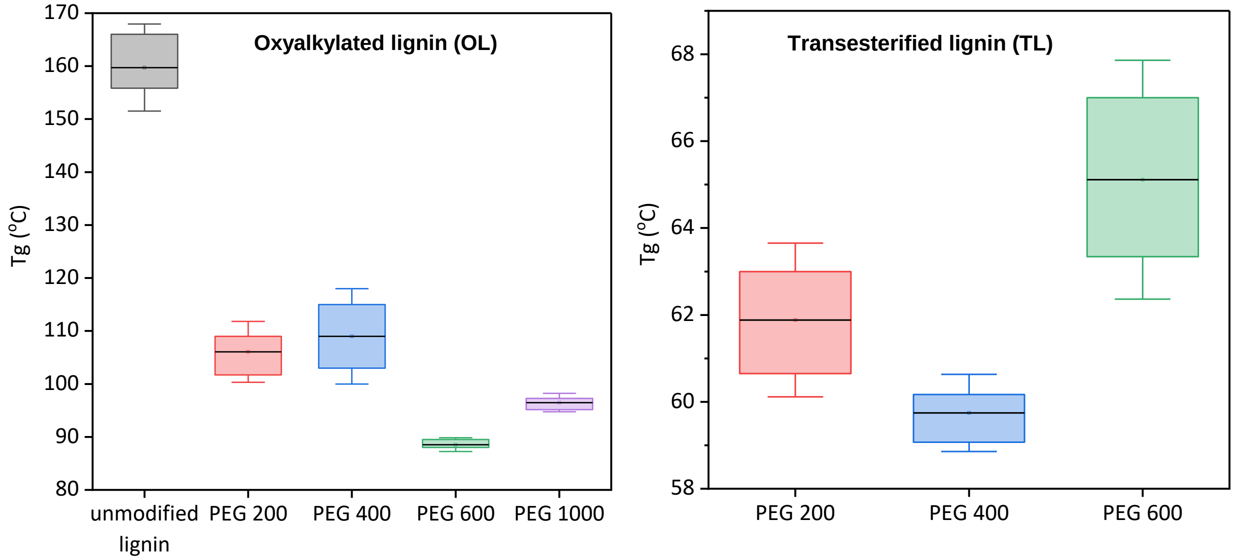

Figure S17: One-way ANOVA ($P \leq 0.05$) statistical analysis of glass transition temperature (*Tg*) of precipitated propylene carbonate oxyalkylated lignin (left) and dimethyl carbonate transesterified lignin (TL)

References

[1] I. Kühnel, B. Saake, R. Lehnen, "Oxyalkylation of lignin with propylene carbonate: Influence of reaction parameters on the ensuing bio-based polyols" *Ind Crops Prod* **2017**, *101*, DOI 10.1016/j.indcrop.2017.03.002.

[2] I. Kühnel, B. Saake, R. Lehnen, "Comparison of different cyclic organic carbonates in the oxyalkylation of various types of lignin" *React Funct Polym* **2017**, *120*, 83–91.

[3] I. Kühnel, Y. Akil, D. Lorenz, B. Saake, R. Lehnen in *NWBC 2018 - Proceedings of the 8th Nordic Wood Biorefinery Conference,* **2018**.

[4] F. R. Vieira, A. Barros-Timmons, D. V. Evtuguin, P. C. O. R. Pinto, "Oxyalkylation of Lignoboost^{TM} Kraft Lignin with Propylene Carbonate: Design of Experiments towards Synthesis Optimization" *Materials* **2022**, *15*, DOI 10.3390/ma15051925.

[5] A.-A. G. Shaikh, S. Sivaram, *Organic Carbonates †*, **1996**.

[6] K. Stokes, R. Mcvenes, J. M. Anderson, "Polyurethane elastomer biostability" *J Biomater Appl* **1995**, *9*, 321–354.

[7] S. Bian, C. Pagan, A. A. Andrianovaartemyeva, G. Du, "Synthesis of Polycarbonates and Poly(ether carbonate)s Directly from Carbon Dioxide and Diols Promoted by a Cs2CO3/CH2Cl2 System" *ACS Omega* **2016**, *1*, 1049–1057.

[8] D. Tang, D. J. Mulder, B. A. J. Noordover, C. E. Koning, "Well-defined biobased segmented polyureas synthesis via a TBD-catalyzed isocyanate-free route" *Macromol Rapid Commun* **2011**, *32*, 1379–1385.

[9] M. Song, X. Yang, G. Wang, "Preparation of polycarbonate diols (PCDLs) from dimethyl carbonate (DMC) and diols catalyzed by KNO3/γ-Al2O3" *RSC Adv* **2018**, *8*, 35014–35022.

[10] J. Sun, D. Kuckling, "Synthesis of high-molecular-weight aliphatic polycarbonates by organo-catalysis" *Polym Chem* **2016**, *7*, 1642–1649.

[11] W. Zhu, X. Huang, C. Li, Y. Xiao, D. Zhang, G. Guan, "High-molecular-weight aliphatic polycarbonates by melt polycondensation of dimethyl carbonate and aliphatic diols: Synthesis and characterization" *Polym Int* **2011**, *60*, 1060–1067.

[12] E. Foy, J. B. Farrell, C. L. Higginbotham, "Synthesis of linear aliphatic polycarbonate macroglycols using dimethylcarbonate" *J Appl Polym Sci* **2009**, *111*, 217–227.

[13] I. Kühnel, J. Podschun, B. Saake, R. Lehnen, "Synthesis of lignin polyols via oxyalkylation with propylene carbonate" *Holzforschung* **2015**, *69*, DOI 10.1515/hf-2014-0068.

[14] L. G. Akim, D. S. Argyropoulos, L. Jouanin, J.-C. Leplé, G. Pilate, B. Pollet, C. Lapierre, "Quantitative 31P NMR spectroscopy of lignins from transgenic poplars" *Holzforschung* **2001**, *55*, 386–390.

[15] D. S. Argyropoulos, “Quantitative phosphorus-31 NMR analysis of six soluble lignins” *Journal of wood chemistry and technology* **1994**, *14*, 65–82.

[16] X. Meng, C. Crestini, H. Ben, N. Hao, Y. Pu, A. J. Ragauskas, D. S. Argyropoulos, “Determination of hydroxyl groups in biorefinery resources via quantitative 31 P NMR spectroscopy” *Nat Protoc* **2019**, *14*, 2627–2647.

[17] D. S. Argyropoulos, “Quantitative phosphorus-31 nmr analysis of lignins, a new tool for the lignin chemist” *Journal of Wood Chemistry and Technology* **1994**, *14*, 45–63.

[18], “Standard Test Method for Dynamic Shock Cushioning Characteristics of Packaging Material 1” **n.d.**, DOI 10.1520/D1596-14.

[19] ASTM Standard D5338-15 (2021), *Standard Test Method for Determining Aerobic Biodegradation of Plastic Materials Under Controlled Composting Conditions Incorporating Thermophilic Temperatures*, **2021**.

[20] International Standard ISO/FDIS 14855-1:2005, *Determination of the ultimate aerobic biodegradability of plastic materials under controlled composting conditions - Method by analysis of evolved carbon dioxide, Part 1: General method*, **2005**.

[21] International Standard ISO/FDIS 14855-2:2007, *Determination of the ultimate aerobic biodegradability of plastic materials under controlled composting conditions - Method by analysis of evolved carbon dioxide, Part 2: Gravimetric measurement of carbon dioxide evolved in a laboratory-scale test*, **2007**.

[22] ASTM, *ASTM D6400 - Standard Specification for Labeling of Plastics Designed to be Aerobically Composted in Municipal or Industrial Facilities*, **2021**.

[23] T. Kijchavengkul, R. Auras, M. Rubino, M. Ngouajio, R. Thomas Fernandez, “Development of an automatic laboratory-scale respirometric system to measure polymer biodegradability” *Polym Test* **2006**, *25*, 1006–1016.
